# Twist-tuned exchange and hysteresis in a bilayer van der Waals magnet

**DOI:** 10.1038/s41467-026-75186-3

**Published:** 2026-07-08

**Authors:** Priyanka Mondal, Sonu Verma, Wenze Lan, Lukas Krelle, Lennard Hopf, Ryan Tan, Regine von Klitzing, Kenji Watanabe, Takashi Taniguchi, Kseniia Mosina, Zdenek Sofer, Akashdeep Kamra, Bernhard Urbaszek

**Affiliations:** 1 Institute for Condensed Matter Physics, TU Darmstadt, Darmstadt, Germany; 2 Department of Physics and Research Center OPTIMAS, Rheinland-Pfälzische Technische Universität Kaiserslautern-Landau, Kaiserslautern, Germany; 3 Institute for High Pressure, Department of Physics, Hanyang University, Seoul, Republic of Korea; 4 Research Center for Electronic and Optical Materials, National Institute for Material Science, Tsukuba, Japan; 5 Research Center for Materials Nanoarchitectonics, National Institute for Material Science, Tsukuba, Japan; 6 Department of Inorganic Chemistry, University of Chemistry and Technology Prague, Prague 6, Czech Republic

**Keywords:** Magnetic properties and materials, Two-dimensional materials

## Abstract

Moiré superlattices in twisted bilayers enable strong reconstruction of electronic band structures, giving rise to correlated phases with high tunability. Extending this concept to van der Waals magnets, we show that twisting induces spatially varying interlayer exchange interactions that can stabilize complex magnetic responses. Here, we demonstrate robust magnetic hysteresis in bilayer CrSBr upon a twist of  ~ 3°, observed as a hysteretic evolution of exciton energies that directly track the underlying magnetic configuration in field-dependent photoluminescence measurements. An analytic two-sublattice model captures this behaviour, attributing it to a twist-induced reduction of interlayer exchange that stabilizes both parallel and antiparallel spin states over a broad field range. Spatially resolved measurements reveal local variations in hysteresis loops, consistent with position-dependent modulation of magnetic parameters. In certain regions, coherent averaging over the moiré unit cell yields an effective monodomain-like response. Our results establish twist engineering as a route to programmable magnetism in two-dimensional antiferromagnets.

## Abstract (web summary)

Twist engineering in van der Waals materials is a simple, though difficult to execute, method for tailoring the electronic band structure. Here, Mondal and coauthors created twisted bilayers of the van der Waals magnet CrSBr and show that small twist angles can be used to control the interlayer exchange interaction.

## Introduction

In two-dimensional (2D) quantum materials, twist engineering has emerged as a powerful technique to control correlated phenomena by deliberately misaligning atomic layers^1–7^. In bilayer graphene and, more recently, bilayer WSe_2_, twisting modifies the electronic band structure, giving rise to correlated insulator phases and superconductivity^8,9^. Here, the spatial coherence of electrons over the larger moiré supercell is essential for the emergence of a new band structure. Employing this approach in van der Waals (vdW) magnets^10,11^ could open new opportunities for spatially programmable magnetism, particularly in systems with layered spin order and antiferromagnetic coupling^12–18^.

Chromium Sulfur Bromide (CrSBr) is a recently identified vdW magnet that combines strong excitonic features with layered antiferromagnetic (AFM) order^19–22^. As an A-type antiferromagnet, CrSBr hosts ferromagnetically aligned spins within each layer, coupled antiferromagnetically between layers^20,23,24^. A unique feature of CrSBr is its unusually strong intralayer ferromagnetic exchange that correlates with its high Néel temperature^22,25^. Beyond magnetism, CrSBr exhibits strong coupling between lattice, electronic, and excitonic degrees of freedom, including exciton-phonon and electron-phonon interactions^26–29^.

## Results and Discussion

**Fig. 1:**
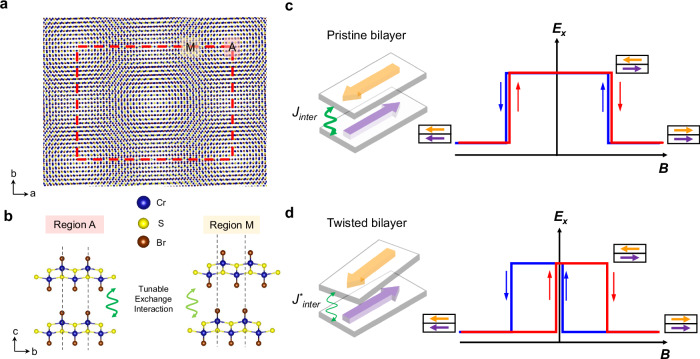
Twist engineering and magnetic hysteresis in bilayer CrSBr. **a** Schematic of the moiré superlattice formed in twisted bilayer CrSBr at a twist angle of *θ* ≈ 3^∘^, with the moiré unit cell outlined by a red dashed box. **b** Side view along the *a*-axis showing two representative atomic registries–A and M–with distinct stacking configurations. These registries modulate the interlayer exchange interaction, resulting in its overall reduction due to averaging over the moiré cell^32^. **c** Schematic of pristine bilayer CrSBr exhibiting spatially uniform interlayer exchange interaction *J*_inter_. The corresponding exciton energy *E*_*x*_, that depends on the angle between the two layer magnetisations^30^, displays negligible hysteresis under an external magnetic field *B* applied along the *b*-axis. Red and blue arrows denote the upward and downward field sweep directions, respectively. **d** In contrast, twisted bilayer CrSBr with moiré-cell-averaged interlayer exchange interaction ${J}_{{{\rm{inter}}}}^{*}$ and magnetic anisotropy^32,33^ exhibits pronounced magnetic hysteresis in the *E*_*x*_-*B* curves. Orange and purple arrows indicate the layer magnetisations.

In bilayer CrSBr, the magnetic configuration, and thus the magneto-optical response, is largely determined by magnetic anisotropies and interlayer exchange interaction, which favours antiparallel alignment of the layer magnetisations^30,31^. Here, we demonstrate that a slight angular misalignment between the two layers of a CrSBr bilayer enables control over the interlayer antiferromagnetic exchange (Fig. 1a, b), in addition to the magnetic anisotropies^32,33^. Consequently, a hysteretic and tunable switching between antiparallel and parallel magnetisation states is achieved (Fig. 1c, d). Figure 1a shows the expected atomic configuration of a twisted bilayer with ~3° twist angle^32^, employed in our experiments. In contrast with the recent large-twist-angle studies in CrSBr^5,17,34,35^, our small-twist sample probes the moiré superlattice and physics^36^ in this system. The interlayer exchange and magnetic anisotropies are expected to be position-dependent within the moiré unit cell^32^ (Fig. 1b). Due to the strong intralayer ferromagnetic exchange, the magnetic state remains spatially homogeneous on the moiré wavelength scales (see Supplementary Notes 1–3). Consequently, our twisted bilayer behaves essentially like its pristine counterpart, but with an effective interlayer exchange ${J}_{{{\rm{inter}}}}^{*}$ obtained by spatial averaging over the moiré cell. Tracking the magnetisation through exciton energy shifts measured by polarization-resolved photoluminescence (PL) provides a highly sensitive probe of the magnetic switching^37–39^.

While the pristine bilayer is found to manifest no magnetic hysteresis (Fig. 1c), the twist-tuned lowering of the interlayer antiferromagnetic exchange makes the parallel magnetisation configuration an energetically stable state for a broader applied field range, resulting in magnetic hysteresis (Fig. 1d) (Supplementary Notes 1 and 2). The good agreement between our experiments and the analytic theoretical model thus exposes a new regime for twisted-bilayer magnets and their magnonic excitations, manifesting coherence over the moiré length scale. This is reminiscent of the similar coherence, which is necessary for the emergence of reduced Brillouin zones and flat bands, in correlated states of electronic matter^1,8^. This regime is enabled by the strong intralayer ferromagnetic exchange in CrSBr and the relatively small moiré wavelength in our sample (Supplementary Note 3). Furthermore, it is complementary to the emergence of spin textures^40^ and formation of spatial domains^41^, which also manifests a different hysteresis^5,12,15,35^. Our demonstrated hysteresis, in sharp contrast, stems from a monodomain Stoner-Wohlfarth switching between antiparallel and parallel magnetisation states that could enable spin filters, memory devices, and reconfigurable logic gates^42,43^ based on antiferromagnetic states.

**Fig. 2:**
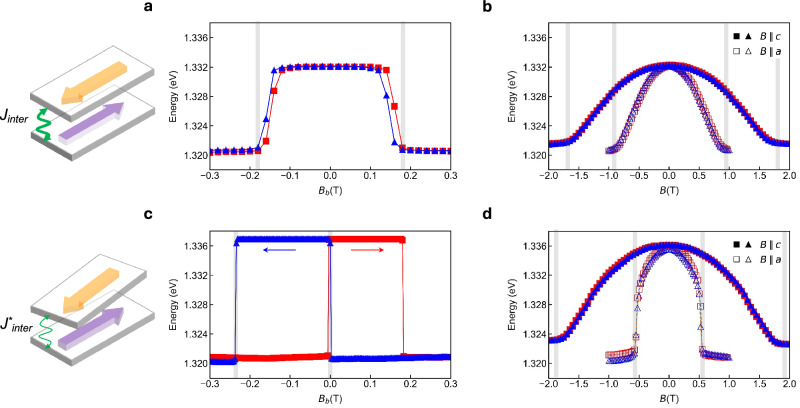
Magnetic field-dependent exciton energy measured via photoluminescence (PL) spectra in twisted and pristine bilayer CrSBr. **a** PL peak energy corresponding to A-exciton measured at T=4.7 K in the pristine bilayer under a magnetic field applied along the in-plane *b*-axis. **b** same as **a** but for a magnetic field applied along the out-of-plane *c*-axis (full symbols) and *a*-axis (hollow symbols). **c** PL peak energy A-exciton of the twisted bilayer under a magnetic field applied along the in-plane *b*-axis. **d** same as **c** but for out-of-plane *c*-axis (full symbols) and *a*-axis (hollow symbols). In all panels, red squares and blue triangles represent magnetic field up and down sweeps, respectively. The measured exciton energy provides direct access to the magnetic configurations (see Fig. 3c) due to the former’s dependence on the angle between the two layer magnetisations. Switching fields are indicated by grey bars.

Using low-temperature (4.7 K) photoluminescence (PL) spectroscopy in a confocal microscope^44^, we probe the magnetic configurations of pristine and twisted bilayer CrSBr through their excitonic responses under applied magnetic fields. The pristine bilayer exhibits a symmetric, reversible PL response, consistent with previous reports^30,31,34^. Figure 2a shows the PL peak energy of the A exciton (see Supplementary Note 4 for spectra) when an in-plane magnetic field is applied along the *b*-axis. Forward and reverse field sweeps, indicated by red squares and blue triangles, are shown for all three directions of the applied magnetic field^45^ (Fig. 2a, b). The A-exciton energy shifts from 1.332 eV to 1.320 eV in both sweep directions, with a switching field of 0.18 T, resulting in only a very narrow hysteresis profile (Fig. 2a).

By contrast, the twisted bilayer shows pronounced hysteresis (Fig. 2c). During the forward sweep, the A-exciton energy redshifts from 1.336 eV to 1.320 eV with a switching field of just 3 mT. In the reverse sweep, a comparable redshift occurs at an even lower switching field of 0 mT, producing a robust hysteresis loop with ferromagnetic order persisting near zero applied field. These stark differences between pristine and twisted bilayers highlight how twist-induced interlayer stacking can stabilize multiple spin states.

To further explore the directional dependence, we applied magnetic fields along the out-of-plane (*c*-axis) and in-plane (*a*-axis) directions (Fig. 2b, d). For the pristine bilayer, applying the field along the *a*-axis shifts the A-exciton energy from 1.332 eV (antiparallel magnetizations) to 1.320 eV (parallel magnetizations) with a switching field of ~0.9 T in both sweep directions, again without hysteresis. Along the *c*-axis, the exciton shifts from 1.332 eV to 1.318 eV with a switching field of  ~ 1.6 T, also with no hysteresis. In the twisted bilayer, applying the field along the *a*-axis produces a shift from 1.336 eV (antiparallel magnetizations) to 1.320 eV (parallel magnetizations) at a switching field of ~0.575 T, with no hysteresis. Along the *c*-axis, the exciton shifts from 1.336 eV to 1.323 eV at ~1.9 T in both directions (see Supplementary Note 5 for details). Thus, hysteresis is observed only in the twisted bilayer for fields along the magnetic easy axis (*b*-axis). Further, the twisted bilayer manifests a magnetic state evolution similar to the pristine sample, but with switching fields altered.

**Fig. 3:**
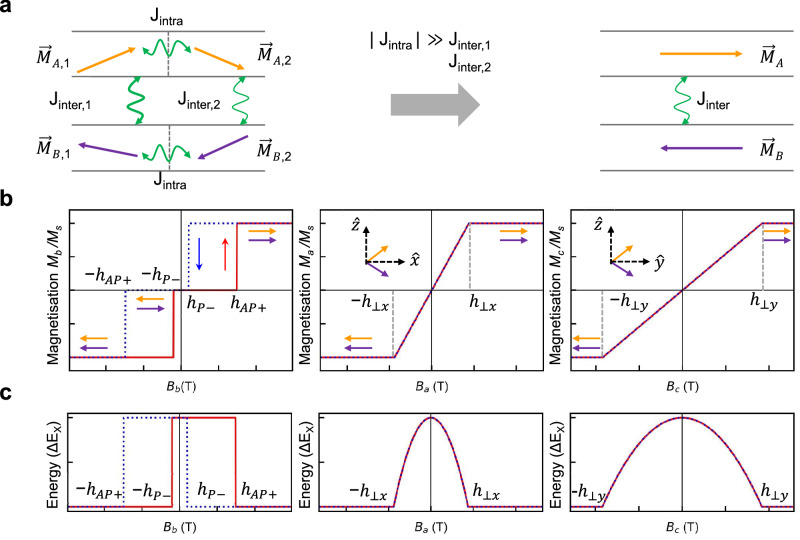
Theoretical analysis of magnetic ground state and hysteresis in a twisted bilayer antiferromagnet. **a** Due to the strong intralayer ferromagnetic exchange, the description of position-dependent interlayer exchange (left) can be effectively reduced to a simplified two-sublattice model (right) with a spatially averaged effective interlayer exchange. **b** Analytically evaluated magnetisation curves *M*/*M*_*s*_ as a function of applied magnetic field along the principal crystal axes: magnetic easy axis *b* (left), intermediate axis *a* (centre), and hard axis *c* (right) respectively. The red solid and blue dotted curves represent up and down magnetic field sweeps, respectively. Here, hysteresis emerges for magnetic field applied along *b* because both parallel and antiparallel magnetic states are stable in the indicated field range. Switching field values are indicated (see main text and Supplementary Note 1) **c** The corresponding exciton energy shift *ΔE*_*X*_ evaluated using the relation $\Delta {E}_{X}\propto {\cos }^{2}\left(\theta /2\right)$ with *θ* being the angle between the two sublattice magnetisations^30,37^.

The twisting of the bilayer magnet produces a complex position-dependent interlayer exchange due to the formation of the moiré unit cell and superlattice^32,33^. However, the intralayer ferromagnetic exchange in CrSBr is much larger than the interlayer exchange and anisotropies, thereby enforcing a spatially homogeneous magnetic state for our twist angle (see Supplementary Note 3). Consequently, for determining the magnetic ground state, the seemingly complex system at hand reduces to a simple two-sublattice model with each layer belonging to one sublattice and the interlayer exchange taking its moiré cell-averaged value [Fig. 3a]. Thus, the same model describes the pristine and twisted bilayers, where the twisting enables a tunability of the interlayer exchange and anisotropies. Here, we disregard the small misalignment between the magnetic anisotropy axes in the two layers of the twisted bilayer, since its effect was found to be negligible for our considered small twist angles. Expressing the magnetic free energy F as^26,46^

1 $$\frac{F}{{M}_{s}}=\,{h}_{E}{{{\boldsymbol{m}}}}_{A}\cdot {{{\boldsymbol{m}}}}_{B}+\frac{{h}_{x}}{2}\left({m}_{Ax}^{2}+{m}_{Bx}^{2}\right)\\+\frac{{h}_{y}}{2}\left({m}_{Ay}^{2}+{m}_{By}^{2}\right)-{{{\boldsymbol{h}}}}_{{{\rm{ext}}}}\cdot \left({{{\boldsymbol{m}}}}_{A}+{{{\boldsymbol{m}}}}_{B}\right),$$

we determine the magnetic state evolution with applied field by evaluating the local minima in the free energy, as detailed in Supplementary Note 1. In Eq. (1), ***m***_*A*_ and ***m***_*B*_ are the unit vectors along A- and B-sublattice magnetisations, *M*_*s*_ is the saturation magnetisation of each sublattice, and we assume Cartesian x, y, z axes to be aligned with the crystal a, c, and b axes. Further, *h*_*E*_, *h*_*x*_, *h*_*y*_, and ***h***_ext_ are *μ*_0_ times the interlayer exchange, intermediate-axis anisotropy, hard-axis anisotropy, and externally applied fields, respectively.

At zero applied field, the magnet is in its antiparallel state with the sublattice magnetisations aligned along the easy (b or z) axis. For applied magnetic field along the intermediate (a or x) or hard (c or y) axes, the magnetisations slowly cant towards the applied field as its strength is increased until they become parallel at a field of *h*_⊥*x*_ = 2*h*_*E*_ + *h*_*x*_ or *h*_⊥*y*_ = 2*h*_*E*_ + *h*_*y*_, respectively (Fig. 3b). For any given field strength for these two axes, only one unique magnetic state is allowed. In sharp contrast, for a field applied along the easy (b or z) axis, there emerges a parameter space and field range that admits both parallel (P) and antiparallel (AP) magnetic configurations as stable states, resulting in hysteresis (see Supplementary Note 1). Consequently, with increasing field strength, the AP configuration is maintained until the field reaches a value denoted as *h*_*AP*+_ and the magnet switches to the P state. On lowering the field, the magnet switches from P to AP at a lower field of *h*_*P*−_ = 2*h*_*E*_ − *h*_*x*_, resulting in hysteresis. The corresponding magnetisation and exciton energy evolution with applied magnetic field along the three axes is depicted in Fig. 3b, c. As detailed in Supplementary Note 1, a canted state also offers a local energy minimum in some range.

If we assume the interlayer exchange in our model to be ferromagnetic, we would only find the P state to minimize the energy for any applied field. Our experiments finding the switching behaviour show that our twisted bilayer sample bears a reduced but clearly antiferromagnetic interlayer exchange, consistent with the expectations based on averaging over the moiré cell^32^. Furthermore, our theoretical analysis provides several switching fields, such as *h*_*P*−_, *h*_⊥*x*_, and *h*_⊥*y*_, which can be read off from the experimental data and employed to determine trends for the effective interlayer exchange as well as anisotropies in the twisted sample, see Supplementary Note 2. Note that our model does not capture microscopic origins such as strain and lattice reconstruction but reproduces the main experimental observations.

Based on our parameter extraction (Supplementary Note 2), we conclude that the experimentally observed switching away from the AP configuration at the field of *h*_*AP*+_ does not agree with our theoretical analysis. Despite good agreement between our theory and experiments on various fronts, this is the only deviation from our model in a wide range of experiments. We presently do not understand the reasons for this. This issue is also pertinent for the pristine bilayer, including for related materials such as CrI_3_, for which our theoretical analysis and established parameters predict a hysteresis that is, however, not observed in experiments^30,31,34^ (Fig. 2a). Thus, besides providing a method for understanding the hysteresis in our twisted bilayers and a reliable extraction of material parameters, our theoretical analysis exposes a key question pertinent to the still broader field of vdW magnets.

**Fig. 4:**
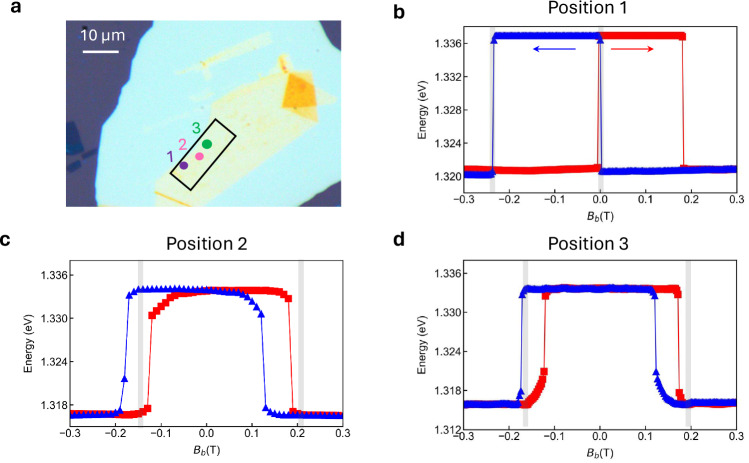
Spatially resolved magneto-optical response of a twisted bilayer magnet. **a** Optical microscope image of the fabricated twisted bilayer sample. The black box indicates the region of interest, with three marked positions (1-3) corresponding to the spatial locations where magneto-optical measurements were performed. Magnetic field-dependent exciton energy measured at positions 1 (**b**), 2 (**c**), and 3 (**d**), under a magnetic field applied along the magnetic easy axis *b*. Red squares represent field sweeps from negative to positive, and blue triangles represent sweeps from positive to negative. The observed hysteresis and energy shifts reflect position-dependent magnetic switching behaviour and interlayer exchange coupling in the twisted bilayer. The switching fields *h*_*P*−_ and *h*_*AP*+_ are indicated by grey lines. While spot 1 manifests only parallel and antiparallel magnetic configurations, spot 2 and spot 3 present evidence for a canted state as well, consistent with our theoretical analysis (Supplementary Note 1).

Our twisted bilayer sample seems to manifest sharp monodomain Stoner-Wohlfarth switching in certain sample locations and does not offer any evidence of domains^35,41^. This is partly because of the large intralayer ferromagnetic exchange in CrSBr. However, due to the inevitable strain and lattice reconstruction effects in twisted bilayer samples, we expect variations in the moiré cell-averaged magnetic interactions over length scales much larger than the moiré wavelength. To examine these spatial variations, we employ position-resolved PL spectroscopy. Figure 4 presents hysteresis loops of the A-exciton energy as a function of the applied magnetic field along the *b*-axis at three representative locations across the sample. Each of the examples shown in Fig. 4b-d exhibits a distinct hysteresis profile, with notable differences in loop width and switching field. The critical field *h*_*P*−_ for forward switching (from negative to positive) varies significantly across positions: ~3 mT at position 1, ~0.14 T at position 2, and ~0.16 T at position 3. The extracted material parameters at each of these positions are presented in Supplementary Note 2. Despite the different switching fields and extracted magnetic parameters, the behaviour at all these positions is consistent with monodomain switching, with indications of the canted state additionally becoming relevant at position 3, as discussed in Supplementary Note 1. This further underscores the usefulness of our simple model.

These spatially dependent features indicate that magnetic switching is influenced by the local variations in the interlayer exchange coupling, ${J}_{{{\rm{inter}}}}^{*}$, and magnetic anisotropies. This tunability can be leveraged to engineer domains with tailored magnetic responses and spatially reconfigurable spintronic devices.

Additionally, for each of the three positions, we measured the A-exciton energy as a function of magnetic field applied along the in-plane *a*-axis and the out-of-plane *c*-axis (see Supplementary Note 7). No hysteresis was observed along these directions, confirming that hysteresis is specific to magnetisation along the magnetic easy axis (*b*-axis), consistent with our theoretical analysis based on twist-controlled average magnetic interactions. To further probe the effect of twisting on magnetic phases in CrSBr, we fabricated a ~2° twisted bilayer-bilayer device (four layers total thickness). By comparing with a pristine four-layer structure, we show in our measurements for the twisted device strong magnetic hysteresis, absent in the pristine layer, confirming its twist-driven origin (see Supplementary Note 8).

*In summary*, we show that introducing a small twist in bilayer CrSBr enables deterministic control of interlayer exchange, leading to controllable magnetic hysteresis that is absent in pristine bilayers. By combining excitonic spectroscopy with an analytic theoretical framework, we establish robust design and characterization principles for moiré magnets via static magnetic configuration studies. Our findings establish twist engineering as a general route to programmable magnetism in van der Waals materials, with direct implications for the design of reconfigurable spintronic devices, excitonic circuits, and monolayer-scale memory technologies.

## Methods

### Sample fabrication

Bulk CrSBr crystals were fabricated through chemical vapour transport^16^. The samples were prepared through mechanical exfoliation onto SiO_2_/Si substrates with an 85 nm SiO_2_ layer. To probe the topology of the CrSBr flakes, an Oxford Instruments Cypher atomic force microscope with AC160 cantilevers was used.

### Atomic force microscopy

Atomic force microscopy measurements were performed at room temperature on a Cypher AFM (Asylum Research/Oxford Instruments, Wiesbaden, Germany). Height images of CrSBr flakes were obtained in AC tapping mode using the cantilever AC160TSA-R3 (300 kHz, 26 N/m, 7 nm tip radius). Images were post-processed with the built-in software features of IGOR 6.38801 (16.05.191, Asylum Research, Santa Barbara, CA, USA).

### Dry transfer

After exfoliating high-quality monolayer CrSBr flakes and hexagonal boron nitride (hBN) flakes onto separate substrates, we employed a dry transfer technique to assemble the twisted heterostructure. The transfer was carried out using a polydimethylsiloxane (PDMS) droplet stamp topped with a thin polypropylene carbonate (PC) film.

The process began with the pickup of the top hBN flake, approximately 30 nm thick. Subsequently, two monolayer CrSBr flakes were sequentially picked up. The pickup of the second CrSBr flake was performed with a rotational misalignment of 3°, controlled via a precision rotator mounted on the optical microscope stage. All CrSBr pickups were conducted at a substrate temperature of 100 °C. Finally, a bottom hBN flake (70 nm thick) was picked up to complete the stack.

The full heterostructure was then released onto a clean Si/SiO_2_ substrate (with 85 nm oxide thickness). To remove the PC film from the top of the stack, the sample was immersed in chloroform for 5 minutes, followed by rinsing in acetone and isopropanol for 3 and 2 minutes, respectively.

### Magneto-optical spectroscopy

Optical spectroscopy was performed using a custom-built confocal setup optimized for magneto-optical measurements^44^. The sample was mounted in a closed-cycle cryostat (AttoDry 1000XL, attocube systems) equipped with a vector magnet capable of applying fields up to 5 T along the out-of-plane *y*-axis (solenoid) and 2 T along the in-plane *x*- and *z*-axes (split coils). Sample positioning relative to a low-temperature achromatic objective was achieved using piezo-positioners (ANPx101 and ANPz102, attocube systems). Photoluminescence (PL) and differential reflectivity (*DR*/*R*) measurements were carried out in backscattering geometry at a base temperature of 4.7 K. The collected signal was spectrally resolved using a Czerny–Turner spectrograph (SpectraPro HRS-500, Teledyne Princeton Instruments) and detected with a CCD camera (Pylon BRexcelon 100, Teledyne Princeton Instruments). For DR/R measurements, a Tungsten–Halogen lamp (SLS201L/M, Thorlabs) was used as a broadband light source, linearly polarized along the crystal *b*-axis using a nanoparticle-film polarizer and an achromatic half-wave plate. For PL, a HeNe laser polarized along the crystal *a*-axis was used for excitation, with emission collected along the *b*-axis. Excitation powers were varied from 600 nW to 240 *μ*W. Magnetic field-dependent measurements were performed by first initializing the CrSBr sample in the FM state, followed by sweeping the magnetic field along different crystallographic directions: from −0.3 T to +0.3 T along the *b*-axis, from −1 T to +1 T along the *a*-axis, and from −2 T to +2 T along the *c*-axis for both the twisted and pristine bilayer. Hysteresis loops were obtained by reversing the sweep direction after reaching the maximum field in each case.

## Supplementary information

Supplementary Information

Transparent Peer Review file

## Data Availability

The data that support the findings of this study are available from the corresponding authors upon request.
