# Supplementary material for: Twist-tuned exchange and hysteresis in a bilayer van der Waals magnet
Source: Nat Commun. 2026 Jul 8;17:5984. doi: 10.1038/s41467-026-75186-3 (PMC13346542; doi:10.1038/s41467-026-75186-3)
Supplement: Supplementary file 1 — Supplementary Information [file 41467_2026_75186_MOESM1_ESM.pdf]

Twist-tuned exchange and hysteresis in a bilayer van der Waals magnet

Supplementary Information

Priyanka Mondal[1], Sonu Verma[2], Wenze Lan[1], Lukas Krelle[1], Lennard Hopf[1], Ryan Tan[1], Regine von Klitzing[1], Kenji Watanabe[3], Takashi Taniguchi[4], Kseniia Mosina[5], Zdenek Sofer[5], Akashdeep Kamra[2],* and Bernhard Urbaszek[1]†

[1]*Institute for Condensed Matter Physics, TU Darmstadt, Hochschulstraße 6-8, D-64289 Darmstadt, Germany*

[2]*Department of Physics and Research Center OPTIMAS, Rheinland-Pfälzische Technische Universität Kaiserslautern-Landau, 67663 Kaiserslautern, Germany*

[3]*Research Center for Electronic and Optical Materials, National Institute for Material Science,1-1 Namiki, Tsukuba 305-0044,Japan*

[4]*Research Center for Materials Nanoarchitectonics, National Institute for Material Science, 1-1 Namiki, Tsukuba 305-0044,Japan and*

[5]*Department of Inorganic Chemistry, University of Chemistry and Technology Prague, Technicka 5, 166 28 Prague 6, Czech Republic*

S1. SUPPLEMENTARY NOTE 1: THEORETICAL DESCRIPTION OF THE MAGNETIC STATE AND HYSTERESIS

In this section, we detail the theoretical framework for describing the magnetic ground state of our twisted CrSBr bilayer as a function of the applied magnetic field along different directions. Our employed two-sublattice model has been successful at describing the pristine bilayer well, while it should be able to describe the magnetization evolution in the twisted bilayer under the assumption that the intralayer ferromagnetic exchange is strong. This assumption is good in CrSBr, but less valid for other van der Waals magnets such as CrI_3, which partly explains our qualitatively different results as compared to the previous studies on CrI_3 [1–3]. Due to the formation of a moiré superlattice in the twisted sample, the interlayer exchange becomes position dependent [?]. Understanding the magnetic ground states for this complex position dependent exchange is a formidable theoretical challenge. As shown below, we are able to capture the main experimental observations by introducing an effective interlayer exchange field in our two-sublattice-model. This effective field stems from a local spatial averaging over the relatively small moiré unit cell for our twisted bilayer magnet.

In subsection S1 A, we summarize our model, mathematical procedure for the evaluation of magnetic states, and our main results. These findings regarding the magnetic state evolution and hysteresis with applied magnetic field strength are further discussed in subsections S1 B, S1 C, and S1 D for the cases of applied field along the easy, intermediate, and hard anisotropy axis, respectively.

A. Summary of the theoretical model and results

Free energy description: We consider a two-sublattice model to describe the antiferromagnetic bilayer. Due to the strong intralayer ferromagnetic exchange in CrSBr [4, 5], the magnetization within each layer is ordered ferromagnetically and represents one sublattice. The interlayer exchange is antiferromagnetic and relatively weak. Thus, it is reasonable to assume that the bilayer twisting by small angles primarily tunes the interlayer antiferromagnetic exchange interaction, although our model allows the anisotropies to vary as well.

* akashdeep.kamra@rptu.de
† bernhard.urbaszek@pkm.tu-darmstadt.de

Further, due to the strong intralayer ferromagnetic exchange, the system is still adequately described as a two-sublattice antiferromagnet with local spatially homogeneous magnetization within each layer.

Within this two-sublattice model, the magnetic free energy F is given by [6, 7]:

$$\frac{F}{M_s} = \mu_0 H_E \boldsymbol{m}_A \cdot \boldsymbol{m}_B + \frac{\mu_0 H_x}{2}(m_{Ax}^2 + m_{Bx}^2) + \frac{\mu_0 H_y}{2}(m_{Ay}^2 + m_{By}^2) - \mu_0 \boldsymbol{H}_{\text{ext}} \cdot (\boldsymbol{m}_A + \boldsymbol{m}_B),$$

$$f = h_E \boldsymbol{m}_A \cdot \boldsymbol{m}_B + \frac{h_x}{2}(m_{Ax}^2 + m_{Bx}^2) + \frac{h_y}{2}(m_{Ay}^2 + m_{By}^2) - \boldsymbol{h}_{\text{ext}} \cdot (\boldsymbol{m}_A + \boldsymbol{m}_B). \tag{1}$$

Here, we have chosen the Cartesian coordinate system in such a way that the x, y, and z axes are respectively along the crystal intermediate (a), hard (c), and easy (b) axes. Consequently, H_x and H_y are positive with $H_y > H_x$ and represent magnetic anisotropy fields along the x and y axes, respectively. H_E is the interlayer exchange field strength, $\boldsymbol{H}_{ext}$ is the externally applied magnetic field, and M_s is the saturation magnetization of each sublattice. $\boldsymbol{m}_{A,B} \equiv \boldsymbol{M}_{A,B}/M_s$ are the unit vectors along the sublattice A and B magnetizations $\boldsymbol{M}_{A,B}$ assumed to be spatially homogeneous. In the equations above, we have simplified the notation by defining $f \equiv F/M_s$ and $h_{E,x,y,\text{ext}} \equiv \mu_0 H_{E,x,y,\text{ext}}$.

Finding free energy minima: Any magnetic state that the system attains in equilibrium corresponds to a magnetization configuration that minimizes the free energy expressed via Eq. (1). It is possible for the system to have multiple local energy minima, in which case hysteresis emerges due to the applied magnetic field history playing a role in the system configuration [8, 9]. This is the standard approach to understanding the hysteresis in a single-domain ferromagnet with uniaxial anisotropy, for example. Thus, we parametrize the magnetic state of our antiferromagnetic bilayer via angles ϕ and β, as shown in Fig. S1 and evaluate their values that minimize the free energy Eq. (1). A local minimum satisfies the conditions [10]:

$$f_\phi = 0,\ f_\beta = 0,\ f_{\phi\phi} > 0,\ f_{\beta\beta} > 0,\ \text{and}\ f_{\phi\phi} f_{\beta\beta} - f_{\phi\beta}^2 > 0, \tag{2}$$

where $f_\beta \equiv \partial f / \partial \beta$, $f_{\phi\phi} \equiv \partial^2 f / \partial \phi^2$ and so on. These conditions provide the equations and inequalities that are solved to obtain the magnetic states that correspond to the local energy minima. Furthermore, we determine the configuration corresponding to the global minimum by comparing the values of the free energy for all the evaluated local minima.

Summary of results: Following the mathematical procedure described above, we evaluate the values of ϕ and β that locally minimize the free energy for a given applied magnetic

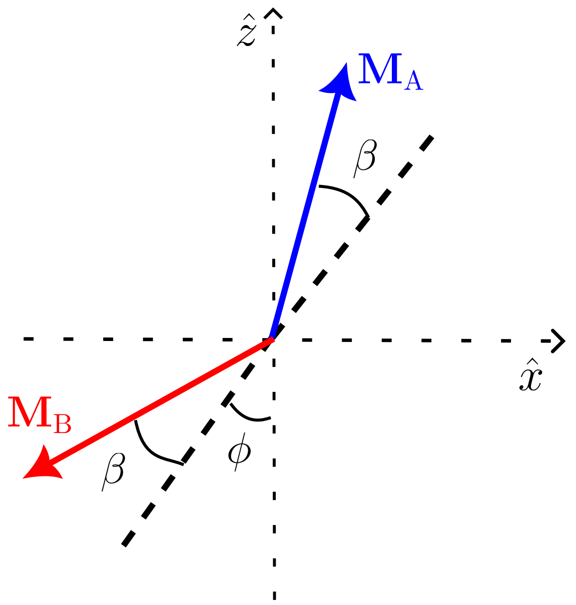

FIG. S1. Schematic depiction of the two sublattice magnetizations' configuration. The different values of angles β and ϕ allow capturing all possible states from antiparallel, parallel, to canted. The schematic depicted adequately captures the cases when the applied field is along the easy ($\hat{z}$) or intermediate ($\hat{x}$) axis. When consider applied field along the hard ($\hat{y}$) axis, the same parametrization of the magnetic configuration works with the x axis replaced by the y axis.

field. At zero applied field, the magnet exists in its expected configuration with antiparallel magnetizations along the z axis.

For the cases of the applied field along the intermediate (subsection S1 C) and the hard (subsection S1 D) axis, we find that with increasing field strength the magnetizations slowly cant towards the applied field direction and become parallel at a large enough field, which is understandably larger for the case of field along the hard axis. However, the system always has a unique configuration that minimizes the energy and thus, there is no hysteresis in these two cases.

When the external field is applied along the easy axis (subsection S1 B), the system remains in the antiparallel (AP) state for low fields. A canted (C) state becomes feasible at somewhat higher fields which evolves into the parallel (P) magnetizations configuration at a large enough field. The unique and remarkable feature, however, is that for a wide range of field values two of the possible three states (AP, C, and P) correspond to local minima

in the free energy. This immediately results in a hysteresis since the system stays in the local energy minimum that it occupies due to the history of applied magnetic field and only switches to a different configuration when the occupied local minimum is eliminated at a specific field strength. As the twisting lowers h_E, the system enters a parameter space that supports a sizable hysteresis between the AP and P states. Furthermore, our theoretical analysis of the possible magnetic configurations allows a direct extraction of the various material parameters, such as interlayer exchange, characterizing the twisted or pristine bilayer employing the experimentally recorded magnetic configurations as a function of the applied field strength.

Anomaly and outstanding challenge: As per our theoretical analysis summarized above and detailed below, hysteresis emerges for the case of magnetic field applied along the easy anisotropy axis. This happens because on increasing the field strength, the system should stay in the AP state as long as it offers a local energy minimum up to an applied field we call h_{AP+} (see Sec. S1 B). On reducing the field, the system stays in the P state until the field is reduced to h_{P-} (Sec. S1 B). As per our and another work's material parameter extraction detailed in Sec. S2, the pristine bilayer should also show this hysteresis because h_{P-} is evaluated to be lower than h_{AP+}. However, we do not find any hysteresis in the pristine bilayer. The same is true for studies on pristine CrI_3 bilayer [1, 2], which is also adequately captured by our two sublattice model. On a careful look, our theoretical model captures all features of the experimental data very well, but just not the observed field at which the system switches away from the AP state.

In summary, our theoretical two-sublattice model predicts hysteresis for both pristine and twisted bilayers. It seems to describe our twisted samples better than the pristine ones, even though the latter have been successfully described using this model for many phenomena. We obtain very good consistence between our theory and experiments if we assume that the experimentally observed field (called h_{AP+} in our theoretical analysis) for switching away from the AP state is somehow not adequately captured by the theory. The reasons for this intriguing anomaly are presently not understood.

Despite this intriguing anomaly, that we leave for future research, our theory and experiment agree very well and allow for an effective extraction of the material parameters for the twisted magnets using the static magnetization data.

Characteristic field	h_{C-}	h_{AP+}	h_{P-}	h_C	h_P
Value in terms of material properties	$h_{P-}\sqrt{\frac{h_x}{2h_E+h_x}}$	$\sqrt{h_x(2h_E+h_x)}$	$2h_E-h_x$	$\sqrt{h_x h_{P-}}$	h_E

TABLE S1. For the case of applied magnetic field along the easy axis, the various characteristic fields that separate the applied field axis into regions with different magnetic states (see Fig. S2) are related to the material properties characterizing the magnetic free energy Eq. (1).

B. Magnetic field applied along the easy axes

Let us consider an applied magnetic field such that $\boldsymbol{h}_{\text{ext}} = h\hat{z}$. Furthermore, assuming the configuration depicted in Fig. S1, the magnetization unit vectors become

$$\boldsymbol{m}_A = \cos(\phi-\beta)\hat{z} + \sin(\phi-\beta)\hat{x},$$
$$\boldsymbol{m}_B = -\cos(\phi+\beta)\hat{z} - \sin(\phi+\beta)\hat{x}. \tag{3}$$

Substituting these in Eq. (1), we obtain the free energy expression f_z relevant for magnetic field applied along the easy axis:

$$f_z = -h_E\cos(2\beta) + \frac{h_x}{2} - \frac{h_x}{2}\cos(2\phi)\cos(2\beta) - 2h\sin\phi\sin\beta. \tag{4}$$

The possible magnetic states are obtained via the values of ϕ and β which correspond to the local minima in the free energy expression Eq. (4) above. This determination of local and global minima in energy is carried out by following the standard procedure and solving the equations outlined in Sec. (S1 A). We directly discuss the results, which are summarized in Fig. S2.

Let us first consider the case when interlayer antiferromagnetic exchange, represented by h_E, is larger than a specific value that we discuss below [Fig. S2(a)]. In this case, the system evolves from being in the antiparallel (AP) state ($\phi = 0, \beta = 0$) to the canted (C) state ($\phi = \pi/2, 0 < \beta < \pi/2$) to the parallel (P) state ($\phi = \pi/2, \beta = \pi/2$), with increasing applied magnetic field h. The AP state presents a local minimum in the free energy for $h < h_{AP+}$ (see Table S1) and the magnet is thus allowed to be in AP state for this range of applied magnetic field. For $h_{C-} < h < h_{P-}$, the C state presents a local minimum and is allowed. Finally, for $h > h_{P-}$, the system is in P state. Interestingly, in the field range $h_{C-} < h < h_{AP+}$, both AP and C present local minima in the free energy. Due to the

multiple allowed states, the system is expected to manifest hysteresis in this field range. The situation is summarized in Fig. S2(a), where the global energy minimum is also represented. This case is relevant when $h_{P-} > h_{AP+}$, which is the case for conventional antiferromagnets with the antiferromagnetic exchange being much larger than the anisotropies. The canted state is described by $\phi = \pi/2$ and

$$\sin\beta = \frac{h}{h_{P-}}. \tag{5}$$

Here, h_{P-} and other characteristic field values discussed above have been defined in Table S1.

The magnetic state evolution is schematically depicted in Fig. S2(b) for the case $h_C < h_{P-} < h_{AP+}$. Here, h_C is the field at which the canted state becomes the global energy minimum with an increasing applied field value. When h_{P-} is further reduced, for example, due to a decrease in the interlayer exchange h_E on account of bilayer twisting, we obtain the state evolution as shown in Fig. S2(c). Now, a new characteristic field h_P emerges. At this value, the P state starts being the global energy minimum with increasing applied field.

The evolution described by Fig. S2(a) is typical for crystalline antiferromagnets (AFMs) which have $h_E \gg h_x$. For our experiments with CrSBr, the situation depicted in Fig. S2 (c) is pertinent, as confirmed by our explicit evaluation of the characteristic fields (Sec. S2) based on the extracted material parameters for pristine as well as twisted bilayers.

Let us consider the hysteretic state evolution observed in our experiments considering Fig. S2 (c). At zero field, we have AP configuration. As the field is increased, the magnet should remain in the AP state until $h = h_{AP+}$ when the AP configuration stops being a local minimum in energy and only P configuration presents a stable state. The magnet remains in P on further increase of the field. Considering the backwards trajectory of lowering the applied magnetic field, the system remains in the P state until the field reaches $h = h_{P-}$ at which P stops presenting a local energy minimum. At this stage, the system can switch to AP or C configurations. Our experiments indicate that it switches to the AP configuration in most cases. This can be rationalized by considering that in going from P to AP configuration, the magnet has to reverse one of the two sublattice magnetizations, which is easier to happen stochastically. For the system to achieve the C state, both sublattice magnetizations would need to be reoriented in a somewhat coordinated fashion, which seems harder to be accomplished by the thermal stochastic forces. Our theoretical model is unable

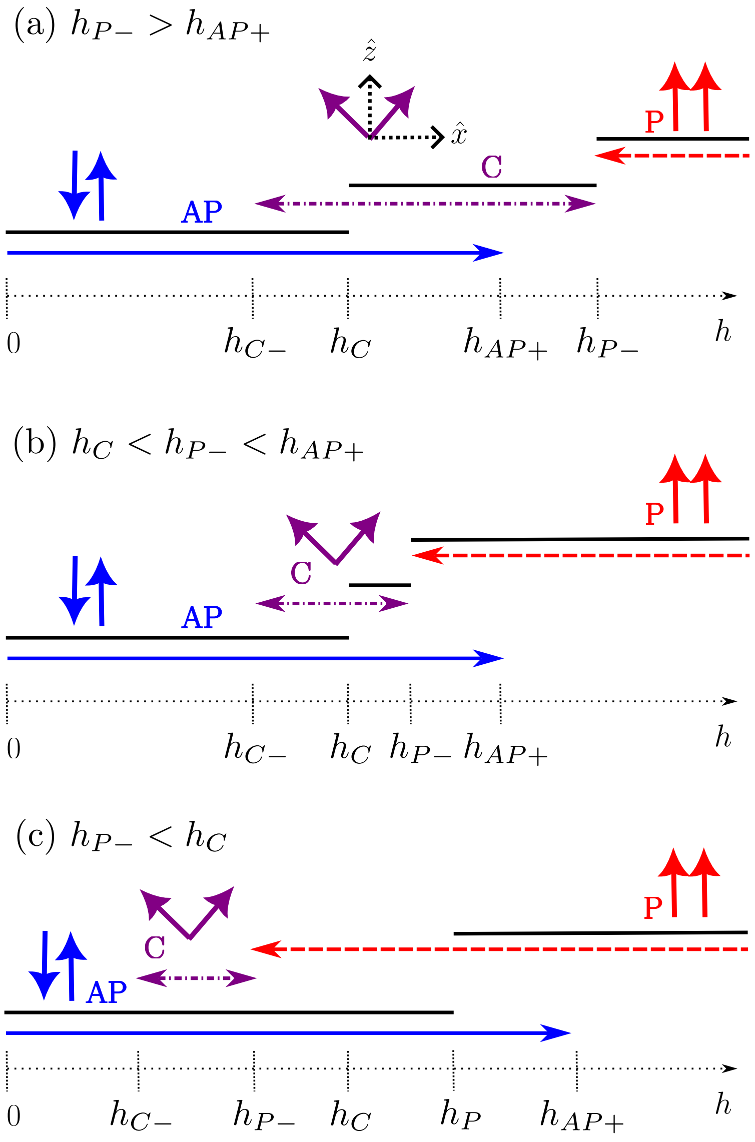

FIG. S2. Schematic depiction of allowed magnetic configurations for a given strength of field h applied along the easy (z) axis. The various characteristic field values are defined in Table S1. Panels (a), (b) and (c) show the situations for three different hierarchies between the characteristic field values. The blue solid, magenta dashed dotted, and red dashed lines depict the presence of antiparallel (AP), canted (C), and parallel (P) magnetic configurations, respectively, as stable allowed states corresponding to local minima in the free energy. The black solid line above highlights the state corresponding to the global minimum in energy for any given applied field h. Magnetic hysteresis emerges when there are multiple stable states for any range of applied field values. Based on the material parameters (Sec. S2), panel (c) is relevant to our CrSBr samples.

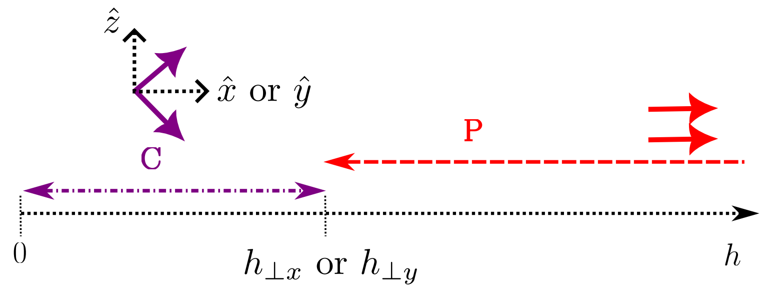

FIG. S3. Schematic depiction of allowed magnetic configurations for a given strength h of the field applied along the intermediate (x) or hard (y) axis. A unique state is allowed for any given field strength. The system is in a canted (C) state until h reaches $h_{\perp x} \equiv 2h_E + h_x$ ($h_{\perp y} \equiv 2h_E + h_y$) for the case of field along intermediate (hard) axis. For larger fields, the system is in parallel (P) state.

to fully capture this stochastic switching, which could be non-deterministic. The state (AP or C) to which the system switches going away from P on lowering the applied field is not deterministic, as discussed above, and we find slightly different behaviors in our data recorded at spots 1, 2, and 3 (see Fig. 4 in the main text). Nevertheless, all these behaviors are anticipated and can be understood within our theoretical model.

As discussed above, our theoretical model expects a certain value (see Table S1) for the field h_{AP+} at which the system switches away from the AP state on increasing the applied field. However, the experiment finds switching at a different value, which is the only inconsistency between our theory and experiment. In other words, our experimentally measured value of h_{AP+} is not consistent with its theoretically evaluated value based on the material parameters (Sec. S2). This anomaly remains even for the case of pristine bilayer where the measured value of h_{AP+} coincides with h_{P-} thereby resulting in no hysteresis. At present, we do not understand the reason for this anomaly. Nevertheless, by acknowledging this unreliability of the measured h_{AP+} and thus disregarding this value, we are still able to extract all material parameters for our pristine and twisted bilayers by comparing theory and experiment, as detailed in Sec. S2.

C. Magnetic field applied along the intermediate axis

Considering the applied magnetic field along the intermediate anisotropy (x) axis, we can repeat the analysis carried out in the previous subsection. The free energy function now takes the form:

$$f_x = -h_E \cos(2\beta) + \frac{h_x}{2} - \frac{h_x}{2} \cos(2\phi) \cos(2\beta) + 2h \cos\phi \sin\beta, \tag{6}$$

in terms of ϕ and β (Fig. S1). Following the procedure described in Sec. S1 A, we minimize the free energy above with respect to ϕ and β thereby obtaining the magnetic states for any given applied field strength h. In this case, we find a unique magnetic configuration for any field value and thus, no hysteresis is expected. The system manifests a canted (C) state with $\phi = \pi$ and

$$\sin\beta = \frac{h}{h_{\perp x}}, \tag{7}$$

for $0 < h < h_{\perp x}$ and the parallel (P) state with $\phi = \pi$ and $\beta = \pi/2$ for $h \geq h_{\perp x}$. Here, $h_{\perp x} = 2h_E + h_x$, and the magnetic state evolution with applied field is schematically summarized in Fig. S3.

D. Magnetic field applied along the hard axis

When the magnetic field is applied along the hard axis, the sublattice magnetizations are no longer pointing in the x-z plane, as was the case in the two cases above. Instead, the applied magnetic field forces the sublattice magnetization to orient in the y-z plane. Nevertheless, we may employ the same parametrization of the general magnetic state as schematically depicted in Fig. S1 with one small change: x axis is replaced by y axis. With this new parametrization, we may follow the same mathematical procedure for obtaining and minimizing the free energy as discussed in the previous section. The obtained result is also nearly identical to the case of magnetic field applied along the intermediate axis (Sec. S1 C) with one key difference: $h_{\perp x}$ is now replaced by $h_{\perp y} \equiv 2h_E + h_y$. The magnetic state evolution is conveniently summarized in Fig. S3 together with the case of field along the intermediate axis.

S2. SUPPLEMENTARY NOTE 2: EXTRACTING TUNED EXCHANGE AND ANISOTROPIES FROM EXPERIMENTAL DATA

Here we provide the procedure and a table to give the field values characterizing our CrSBr samples extracted from experiments.

Measured switching fields and error analysis: The measured switching fields h_{P-}, h_{AP+}, $h_{\perp x}$ and $h_{\perp y}$ for pristine and twisted bilayer (Spot 1) are marked in the Figure 2 of the main text. The switching fields of twisted bilayer Spot 2 and 3 are shown in Figure 4 of the main text and in Figure S12 and S14. The measurements were performed with a magnetic field stepsize of 10 mT along the b-axis. But for extracting the critical fields, in particular for Spot 2 and 3 of the twisted sample, the error is larger, as the change in magnetisation does not occur as a step-like function. For the a and c axis the error bars can be set equal to the magnetic field step-size. We therfore estimate the error of extracted fields to be ±15 mT, ±25 mT, ±50 mT under the external magnetic fields parallel to the b, a, and c axis, respectively.

Extracting material properties: We have four measured fields - h_{P-}, h_{AP+}, $h_{\perp x}$ and $h_{\perp y}$, and only three variables to be determined - h_E, h_x, and h_y. Thus, the system of equations is overdetermined. On careful analysis, we find that employing the following expressions

$$h_{P-} = 2h_E - h_x, \tag{8}$$

$$h_{\perp x} = 2h_E + h_x, \tag{9}$$

$$h_{\perp y} = 2h_E + h_y, \tag{10}$$

and solving for the three desired field values (h_E, h_x, and h_y) yields a good procedure to extract these. We find that employing the theoretical expression for h_{AP+} (Table S1) in the extraction procedure produces unphysical and anomalous field values, which do not compare at all with other determinations in the literature. This, and several other indications, convince us that the experimentally recorded value of h_{AP+} does not correspond to its theoretical evaluation within our model for reasons that we do not understand at present. Furthermore, in order to validate our procedure for extracting the field values characterizing the material, we compare our results with the previous results on bulk CrSBr [7]. These are also reported and discussed in the table below and we find a good agreement. We further note that the use of ferromagnetic resonance frequencies for fitting and extracting the

field values in Ref. [7] produces some deviation from our extracted values. If we use the static measurements from Ref. [7] to extract the fields characterizing the material, we get a better agreement with our extracted values. At the same time, this shows that fitting the ferromagnetic resonance frequencies data and static magnetization data using the same free energy produces slightly different extracted values of the fields characterizing the material.

TABLE S2. Extraction of fields (in Tesla) characterizing the material from experimental data.

	Measured				Extracted			Evaluated		
Sample	h_{P-}	h_{AP+}	$h_{\perp x}$	$h_{\perp y}$	h_x	h_y	h_E	h_{AP+} [i]	h_{C-} [ii]	h_C [iii]
Twisted (Spot 1)	0	0.237	0.575	1.900	0.288	1.612	0.144	0.407	0	0
Twisted (Spot 2)	0.140	0.210	0.500	1.800	0.180	1.480	0.160	0.300	0.084	0.159
Twisted (Spot 3)	0.160	0.250	0.600	1.800	0.220	1.420	0.190	0.363	0.097	0.188
Pristine bilayer	0.180	0.180	0.900	1.600	0.360	1.060	0.270	0.569	0.114	0.255
Bulk (Ref. [7]) [iv]	N/A				0.383	1.30	0.395	0.670	0.233	0.395

[i] $h_{AP+} = \sqrt{h_x(2h_E + h_x)}$ (Table S1)

[ii] $h_{C-} = h_{P-}\sqrt{\frac{h_x}{2h_E+h_x}}$ (Table S1)

[iii] $h_C = \sqrt{h_x h_{P-}}$ (Table S1)

[iv] The extracted values of the fields here have been taken directly from Ref. [7]. These field values were extracted by fitting the ferromagnetic resonance data at 5K to the corresponding theoretical calculation based on the same free energy that we have employed [Eq. 1] in our analysis of the magnetic ground state and hysteresis.

S3. SUPPLEMENTARY NOTE 3: MOIRÉ LENGTH SCALES AND DOMAIN-WALL FORMATION CRITERIA IN TWISTED BILAYER CRSBR

In this section, we provide an energetic estimate for (i) the width $\delta^\star$ of a 180° interlayer domain wall and (ii) the critical twist angle θ_c above which the formation of moiré-scale AF regions becomes energetically unfavorable in twisted bilayer *CrSBr*. The estimate relies on a minimal two-layer description: the intralayer ferromagnetic exchange $J_\parallel$ sets the spin stiffness (gradient cost), while local anisotropy and interlayer exchange set the potential-energy cost inside the wall. For twist angles of order a few degrees (e.g., $\theta \simeq 3°$), the CrSBr wall width is comparable to or larger than the moiré length ($\delta^\star/L_M \gtrsim 1$) and θ lies above the estimated crossover θ_c; hence, the formation of moiré-scale AF/FM domains separated by narrow walls is energetically unfavorable at $\theta \simeq 3°$, and the magnetic response is expected to remain essentially collinear (well described by an effective, spatially averaged interlayer exchange). For comparison, we report the corresponding numbers for twisted bilayer CrI_3 within the same framework [11–13].

Moiré length scales. For a small twist angle θ (in radians), the moiré lengths are

$$a_M = \frac{a}{2\sin(\theta/2)} \approx \frac{a}{\theta} \qquad (\mathrm{CrI_3}), \tag{11}$$

$$L_a = \frac{a_0}{2\sin(\theta/2)}, \qquad L_b = \frac{b_0}{2\sin(\theta/2)} \qquad (\mathrm{CrSBr}), \tag{12}$$

$$L_M \equiv \sqrt{A_M} \approx \sqrt{L_a L_b} \approx \frac{\sqrt{a_0 b_0}}{\theta}. \tag{13}$$

We use $\ell_M = a_M$ for CrI_3 and $\ell_M = L_M$ for CrSBr.

A. Domain-wall energy and width.

We consider two coupled magnetic layers with strong intralayer ferromagnetic exchange $J_\parallel > 0$ and a stacking-dependent interlayer coupling. In an AF-favored fraction f_{AF} of a moiré unit cell, the local interlayer energy per spin is taken as

$$\varepsilon_\perp(\phi) = J_\perp\big(1 + \cos\phi\big), \tag{14}$$

where ϕ is the local interlayer angle ($\phi = 0$ FM and $\phi = \pi$ AF) and $J_\perp > 0$.

CrI_3 is described with uniaxial easy-axis anisotropy $\varepsilon_K^{(\mathrm{CrI_3})} = K_z(1 - m_z^2) = K_z \sin^2\phi$ [11,

13]. CrSBr is modeled with biaxial anisotropy

$$\varepsilon_K^{(\mathrm{CrSBr})} = \frac{K_x}{2} m_x^2 + \frac{K_y}{2} m_y^2, \qquad (K_y > K_x > 0), \tag{15}$$

consistent with triaxial anisotropy measurements [14]. The lowest-cost 180° wall avoids the hard axis and lies in the xz plane ($m_y = 0$), such that the relevant anisotropy scale is K_x.

A wall of width δ traversing a moiré unit cell of linear size ℓ_M occupies area $\sim \ell_M \delta$ and contains

$$N_{\mathrm{DW}} \sim \frac{\ell_M \delta}{a_{\mathrm{eff}}^2} \tag{16}$$

spins, where a_{eff} sets the areal spin density ($a_{\mathrm{eff}} = a$ for CrI_3 and $a_{\mathrm{eff}} \simeq \sqrt{a_0 b_0}$ for CrSBr).

Gradient (intralayer exchange) contribution. Across the wall, the angle changes by $\Delta\phi \simeq \pi$ over distance δ, giving a nearest-neighbor difference $\Delta\phi_{\mathrm{nn}} \sim \pi a_{\mathrm{eff}}/\delta$. For small $\Delta\phi_{\mathrm{nn}}$, the intralayer exchange penalty per bond scales as $\sim (J_\parallel/2)\Delta\phi_{\mathrm{nn}}^2$, which yields

$$E_\parallel(\delta) \sim N_{\mathrm{DW}} \frac{J_\parallel}{2} \left(\frac{\pi a_{\mathrm{eff}}}{\delta}\right)^2 \approx \frac{\pi^2}{2} \left(\frac{\ell_M}{\delta}\right) J_\parallel. \tag{17}$$

Local contribution inside the wall (anisotropy + interlayer exchange). Approximating the wall as uniformly sampling $\phi \in [0, \pi]$ gives $\langle \sin^2\phi \rangle = 1/2$ and $\langle 1 + \cos\phi \rangle = 1$. Writing the wall-relevant anisotropy as

$$K_{\mathrm{loc}} = \begin{cases} K_z, & \mathrm{CrI_3}, \\ K_x/2, & \text{CrSBr (wall in the } xz \text{ plane with the convention above)}, \end{cases} \tag{18}$$

the mean local penalty per wall spin becomes $\langle \varepsilon_K \rangle + \langle \varepsilon_\perp \rangle = K_{\mathrm{loc}}/2 + J_\perp$, hence

$$E_{\mathrm{loc}}(\delta) \approx \left(\frac{\delta\, \ell_M}{2a_{\mathrm{eff}}^2}\right) (K_{\mathrm{loc}} + 2J_\perp). \tag{19}$$

Combining both contributions gives the approximate wall energy per moiré unit cell:

$$E_{\mathrm{DW}}(\delta) \approx \frac{\pi^2}{2} \left(\frac{\ell_M}{\delta}\right) J_\parallel + \left(\frac{\delta\, \ell_M}{2a_{\mathrm{eff}}^2}\right) (K_{\mathrm{loc}} + 2J_\perp). \tag{20}$$

Optimized wall width. Minimizing Eq. (20) with respect to δ yields

$$\delta^\star \approx \pi a_{\mathrm{eff}} \sqrt{\frac{J_\parallel}{K_{\mathrm{loc}} + 2J_\perp}}, \tag{21}$$

and the corresponding minimized wall energy cost

$$E_{\mathrm{DW}}^\star \approx \pi \left(\frac{\ell_M}{a_{\mathrm{eff}}}\right) \sqrt{J_\parallel (K_{\mathrm{loc}} + 2J_\perp)}. \tag{22}$$

B. AF-domain energy gain and critical angle.

An AF-favored region of area fraction $f_{\rm AF}$ contains $N_M \sim f_{\rm AF}(\ell_M/a_{\rm eff})^2$ spins per moiré unit cell. Converting this region from FM ($\phi = 0$) to AF ($\phi = \pi$) lowers the interlayer energy per spin by $2J_\perp$, giving

$$E_{\rm AF} \approx 2f_{\rm AF}\left(\frac{\ell_M}{a_{\rm eff}}\right)^2 J_\perp. \tag{23}$$

A textured state is favored when $E_{\rm AF} \gtrsim E^\star_{\rm DW}$, which implies

$$\frac{a_{\rm eff}}{\ell_M} \approx \frac{2}{\pi}\frac{f_{\rm AF}J_\perp}{\sqrt{J_\parallel(K_{\rm loc}+2J_\perp)}}. \tag{24}$$

Using $a_{\rm eff}/\ell_M \propto \theta$ (Eqs. (11)–(13)) gives θ_c up to order-one geometry factors. Following the convention of Ref. [11], we also quote the commonly used scaling form in which the explicit factor $2/\pi$ is absorbed into the numerical prefactors of the wall estimate:

$$\theta_c \approx \frac{f_{\rm AF}J_\perp}{\sqrt{J_\parallel(K_{\rm loc}+2J_\perp)}}. \tag{25}$$

C. Material parameters and numerical estimates.

CrSBr. We use representative low-temperature values consistent with Refs. [14, 15]:

$$\begin{aligned} J_\parallel &= 3.88\ \text{meV},\ J_\perp = 0.0282\ \text{meV},\ K_x = 0.016\ \text{meV},\ K_y = 0.034\ \text{meV},\\ a_{\rm eff} &= \sqrt{a_0 b_0} = 0.408\ \text{nm}, \end{aligned} \tag{26}$$

with $a_0 = 3.50$ Å and $b_0 = 4.76$ Å. For CrSBr, $\ell_M = L_M$ and $K_{\rm loc} = K_x/2$. Equation (21) gives $\delta^\star_{\rm CrSBr} \approx 9.95$ nm. If the biaxial anisotropy is instead written as $\varepsilon_K = K_x m_x^2 + K_y m_y^2$ (without the explicit factor 1/2), then $K_{\rm loc}$ doubles and $\delta^\star_{\rm CrSBr} \approx 9.38$ nm. For $f_{\rm AF} = 0.5$, Eq. (25) gives $\theta_c^{(\rm CrSBr)} \approx 1.62°$, while Eq. (24) gives $\theta_c^{(\rm CrSBr)} \approx 1.03°$.

CrI_3 (comparison). We use representative low-temperature values consistent with Ref. [11] and stacking-dependent interlayer-exchange analysis [13]:

$$J_\parallel = 2.00\ \text{meV},\quad J_\perp = 0.100\ \text{meV},\quad K_z = 0.300\ \text{meV},\quad a_{\rm eff} = a = 0.687\ \text{nm}. \tag{27}$$

For CrI_3, $K_{\rm loc} = K_z$ and $\ell_M = a_M$. Equation (21) gives $\delta^\star_{\rm CrI_3} \approx 4.32$ nm. For $f_{\rm AF} = 0.5$, Eq. (25) gives $\theta_c^{(\rm CrI_3)} \approx 2.865°$, while Eq. (24) gives $\theta_c^{(\rm CrI_3)} \approx 1.824°$, consistent with the disappearance of a textured state above $\sim 3°$ [11, 12].

TABLE S3. Critical twist angle θ_c (degrees) versus $f_{\rm AF}$ in the present estimate. "Scaling form" uses Eq. (25); "with $2/\pi$" uses Eq. (24).

$f_{\rm AF}$	CrI_3 (scaling form)	CrI_3 (with $2/\pi$)	CrSBr (scaling form)	CrSBr (with $2/\pi$)
0.0	0.000	0.000	0.000	0.000
0.1	0.573	0.365	0.323	0.206
0.2	1.146	0.730	0.646	0.412
0.3	1.719	1.094	0.970	0.617
0.4	2.292	1.459	1.293	0.823
0.5	2.865	1.824	1.616	1.029
0.6	3.438	2.189	1.939	1.235
0.7	4.011	2.553	2.263	1.440
0.8	4.584	2.918	2.586	1.646
0.9	5.157	3.283	2.909	1.852
1.0	5.730	3.648	3.232	2.058

TABLE S4. Moiré length scales and domain-wall-to-moiré ratios. For CrSBr we use $L_M = \sqrt{L_a L_b}$ and report $\delta^\star_{\rm CrSBr}/L_M$; for CrI_3 we report $\delta^\star_{\rm CrI_3}/a_M$.

θ (deg)	a_M (CrI_3) [nm]	L_a (CrSBr) [nm]	L_b (CrSBr) [nm]	$\delta^\star_{\rm CrI_3}/a_M$	$\delta^\star_{\rm CrSBr}/L_M$
0.5	78.72	40.11	54.55	0.055	0.213
1.0	39.36	20.05	27.27	0.110	0.425
2.0	19.68	10.03	13.64	0.220	0.851
3.0	13.12	6.68	9.09	0.329	1.277

D. Domain-wall width relative to the moiré length.

Twist mainly changes the moiré lengths ($\propto \theta^{-1}$), while $\delta^\star$ is set by microscopic couplings. A practical criterion for whether a well-defined domain pattern can fit inside the moiré cell is $\delta^\star/L_M \ll 1$ (narrow domain walls relative to the moiré length). Table S4 lists representative moiré lengths and ratios at selected twist angles

For CrSBr at $\theta \simeq 3^\circ$. Table S4 gives $L_M \simeq \sqrt{6.68 \times 9.09}\,\mathrm{nm} \approx 7.8\,\mathrm{nm}$ and $\delta^\star/L_M \simeq$

1.28 at 3°. Thus, domain walls are not narrow on the moiré scale, which disfavors well-separated AF/FM domains and supports a coherently averaged interlayer exchange picture (monodomain switching).

S4. SUPPLEMENTARY NOTE 4: PRISTINE BILAYER

Supplementary Note S4 illustrate thes structural, optical, and magneto-optical properties of pristine bilayer CrSBr, as we compare in the main text the pristine with a twisted bilayer (see Supplementary Note S5). The crystal structure along multiple axes, highlighting the layered arrangement of Cr, S, and Br atoms and the anisotropic magnetic ordering within and between layers is shown in Fig. S4. The photoluminescence (PL) spectrum at 4.7 K and zero magnetic field of pristine bilayer CrSBr in Fig. S5 shows two exciton species. The peaks located at 1.332 eV and 1.37eV are assigned to the A and B exciton, respectively, consistent with previous reports[16, 17]. Detailed magneto-optical measurements under applied magnetic fields along the three crystallographic axes demonstrate the evolution of the A-exciton intensity as the system transitions between antiferromagnetic and ferromagnetic regimes in Fig. S6, for details of the vector magnet set-up see [18]. We do not observe hysteresis of the magnetization for magnetic field sweeps for the pristine bilayer.

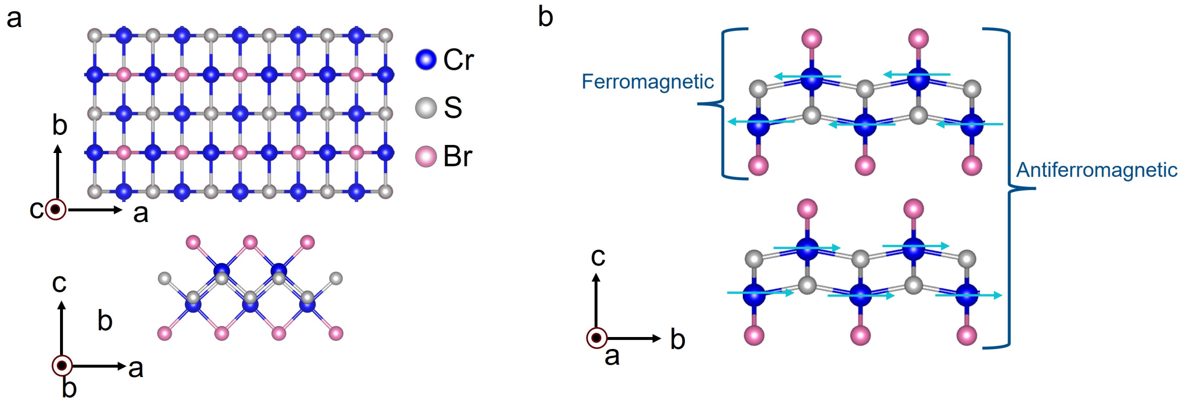

FIG. S4. **Crystal structure of CrSBr.** (a) Crystal structure oriented along the c- and b-axes, showing the layered arrangement of Cr, S, and Br atoms. (b) Projection along the a-axis, highlighting the in-plane magnetic spin alignment. Within a single layer, the Cr atoms exhibit ferromagnetic ordering, while adjacent layers are antiferromagnetically coupled. This layered magnetic structure underlies the anisotropic magnetic and magneto-optical properties of CrSBr.

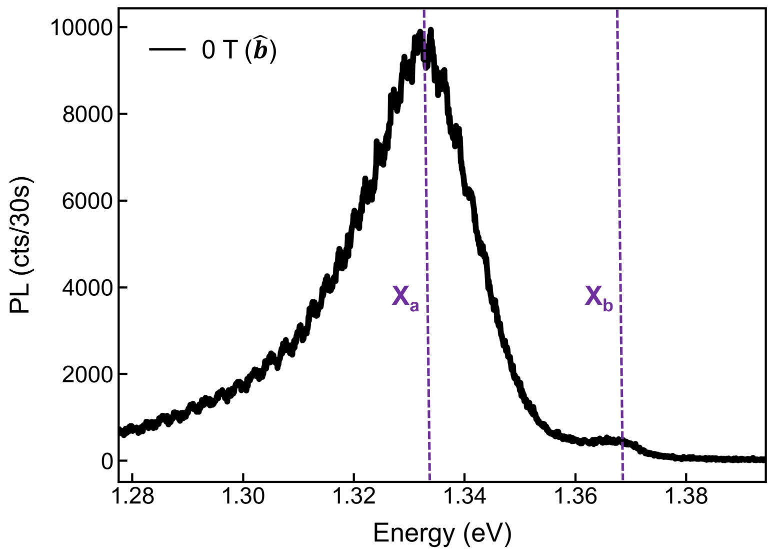

FIG. S5. **Photoluminscence spectrum of pristine bilayer CrSBr.** Two excitonic species, the A and B excitons, are observed in the spectra of pristine bilayer CrSBr at 0 T and 4.7 K, appearing at energies of 1.332 eV and 1.367 eV, respectively.

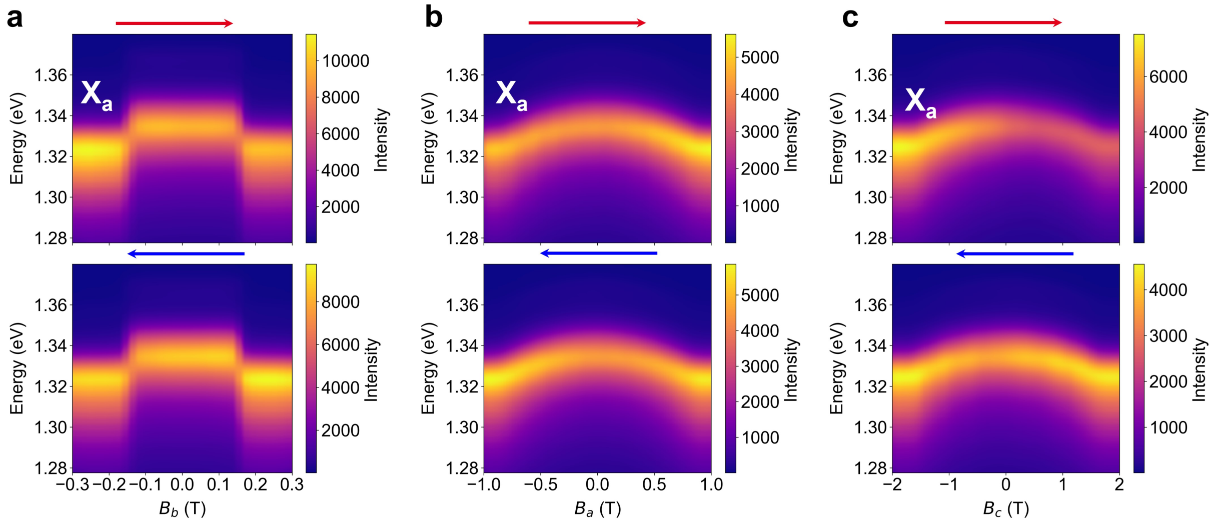

FIG. S6. **Magneto-optical measurements on the pristine bilayer under magnetic fields applied along specific crystallographic directions.** Panels **a**, **b**, and **c** show the magnetic field dependence along the b-, a-, and c-axes, respectively. For each orientation, the top row displays the data for increasing field (from negative to positive), and the bottom row for decreasing field (from positive to negative). The A-exciton becomes brighter as the system transitions from the antiferromagnetic to ferromagnetic regime.

S5. SUPPLEMENTARY NOTE 5: EXCITONIC TRANSITIONS IN TWISTED BILAYER CRSBR.

In the twisted bilayer at B= 0 T and T= 4.7 K, we newly observe distinct peaks in PL at 1.336 eV and 1.37 eV. Both transitions are also clearly visible in differential reflectance spectra DR/R (Fig. S7), indicating their high oscillator strength[19]. Both the PL and reflectivity spectra exhibit two clear excitonic features, indicating consistent optical responses from the twisted bilayer structure.

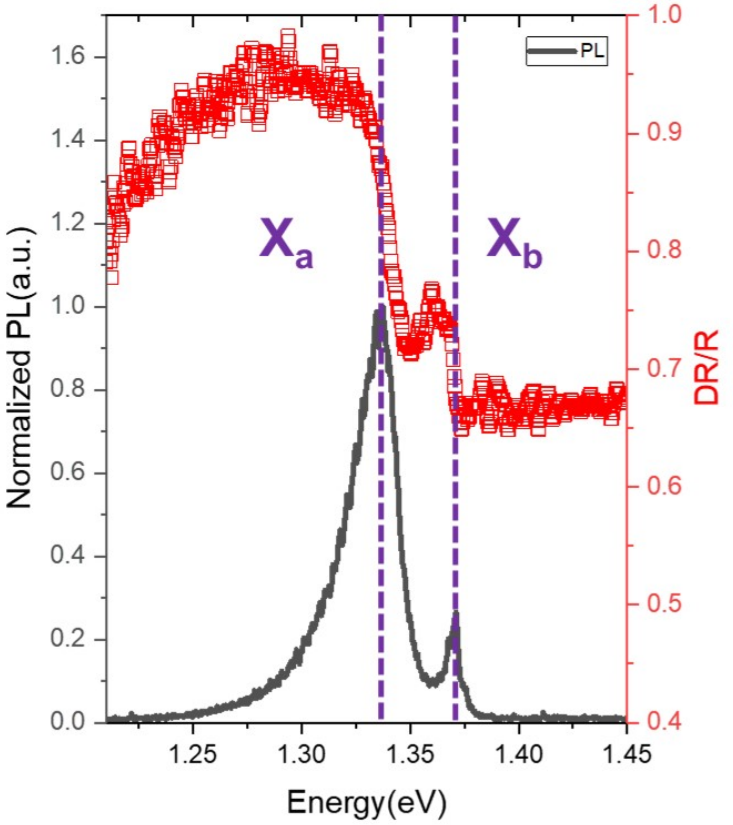

FIG. S7. **Twisted bilayer photoluminescence and differential reflectance(DR/R) measurements at 4.7 K at zero applied magnetic field.** The black curve represents the photoluminescence (PL) spectrum of the twisted bilayer, showing two distinct peaks corresponding to the A-exciton at 1.336 eV and the B-exciton at 1.370 eV. The red curve shows DR/R measured at the same sample position.

We examined the polarization properties of these excitons in both the ferromagnetic (±0.3 T along b) and antiferromagnetic (0 T) states. Regardless of the spin configuration,

both excitons remain strongly polarized along the b crystallographic axis (Fig. S8).

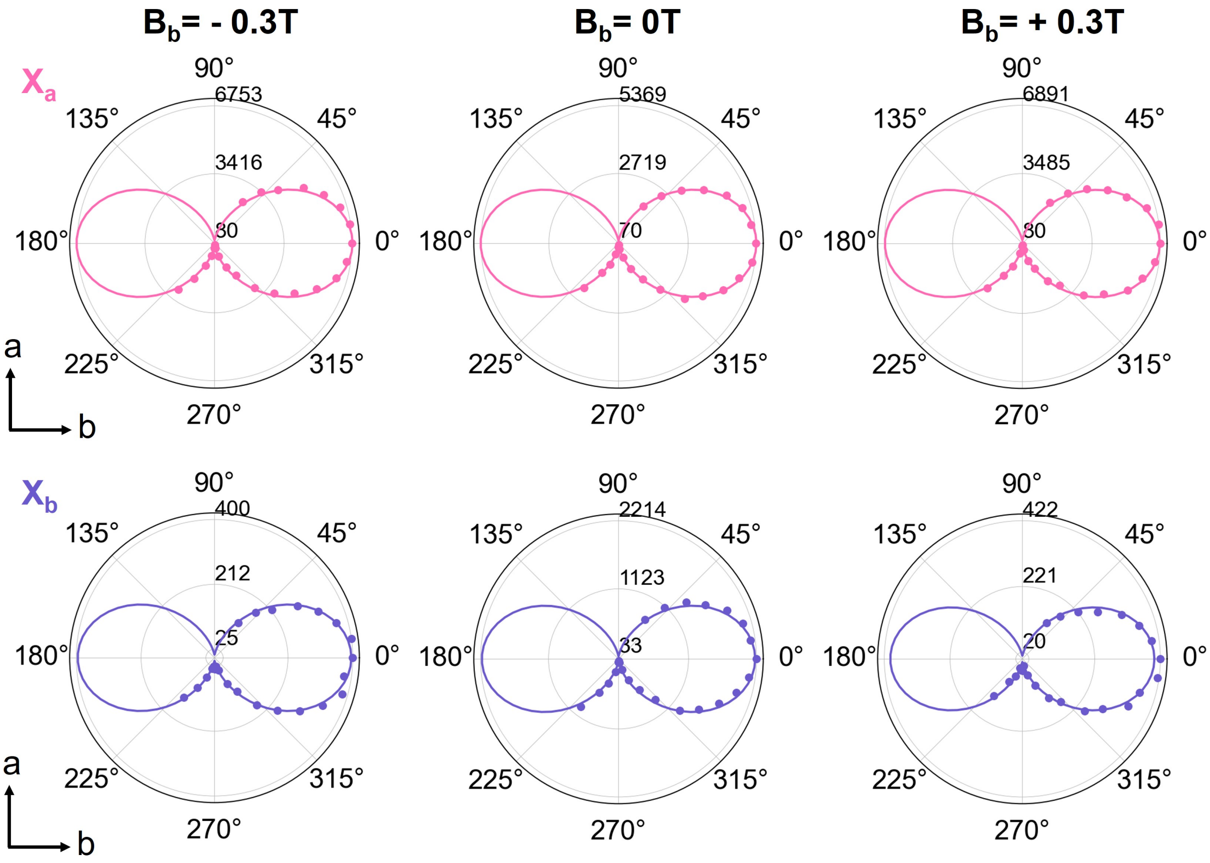

FIG. S8. **Polarization-resolved photoluminescence (PL) from the twisted bilayer sample at T=4.7 K using a half-wave plate (HWP) to change the excitation polarization direction.** Top panel shows the polarization dependence of A-exciton and bottom panel of the B-Exciton. The left panel shows the polarization-resolved PL at a magnetic field of –0.3 T along the b-direction, the middle panel corresponds to zero field, and the right panel shows the measurement at +0.3 T along the b-direction. Pink dots represent the A-exciton, while purple dots correspond to the B-exciton.

In terms of PL intensity, the A and B excitons show contrasting behavior. The B exciton becomes dimmer in the ferromagnetic state, while the A exciton becomes brighter compared to the antiferromagnetic configuration, see axis labels in (Fig. S8). This trend is consistently observed during magnetic field sweeps along all three principal crystallographic directions (Fig. S9).

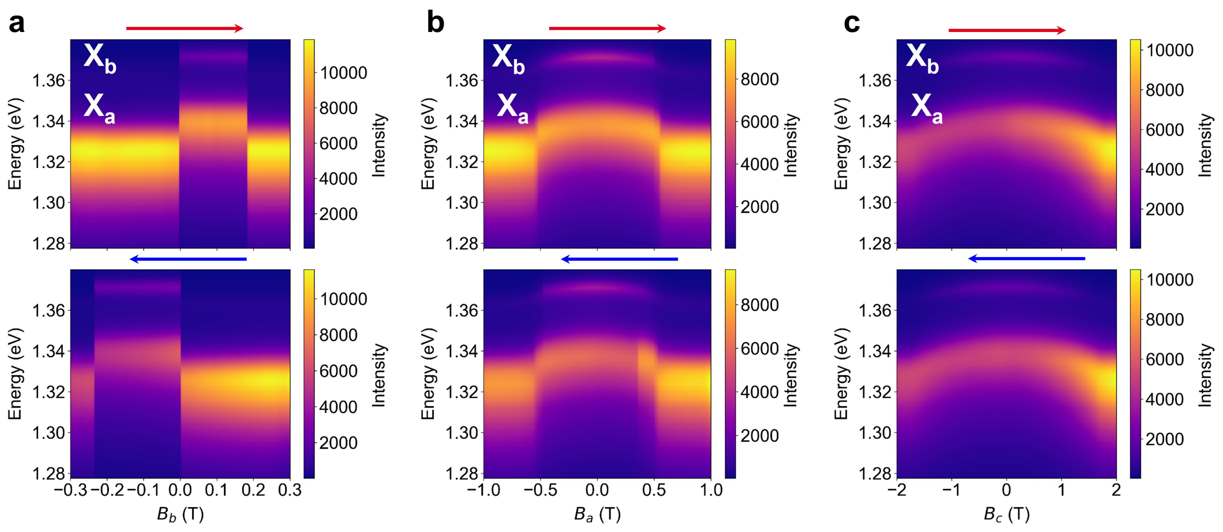

FIG. S9. **Magneto-optical measurements on the twisted bilayer at spot 1 under magnetic fields applied along specific crystallographic directions.** **a** shows the magnetic field dependence along the b-axis, **b** corresponds to the a-axis, and **c** represents the c-axis. In each case, the top row for each figure shows data for increasing magnetic field (negative to positive), while the bottom row shows the response for decreasing field (positive to negative).

S6. SUPPLEMENTARY NOTE 6: EVOLUTION AND REPRODUCIBILITY OF THE HYSTERESIS.

Supplementary Note S6 shows the evolution of the magnetic hysteresis curves for the A-exciton as the maximum applied magnetic field (B_{max}) is increased along the b-direction. The curves are displayed vertically from bottom to top, corresponding to B_{max} = 0, 0.1, 0.2, and 0.3 T. In each measurement, the magnetic field is swept from –0.3 T up to the designated B_{max} and then back to –0.3 T, completing a full cycle. This sequential evolution reveals how the hysteresis loop develops with increasing field: the loop gradually widens and its shape changes, directly reflecting the progressive reorientation and modification of the underlying magnetic states within the bilayer. These results highlight the sensitivity of the excitonic magneto-optical response to the strength of the applied magnetic field and show reproducibility of the hysteretic behavior. We carried out the same loop measurements at spot 3 and we see switching between 2 magnetization states in a fine scan with 3 mT steps, see Fig.S11.

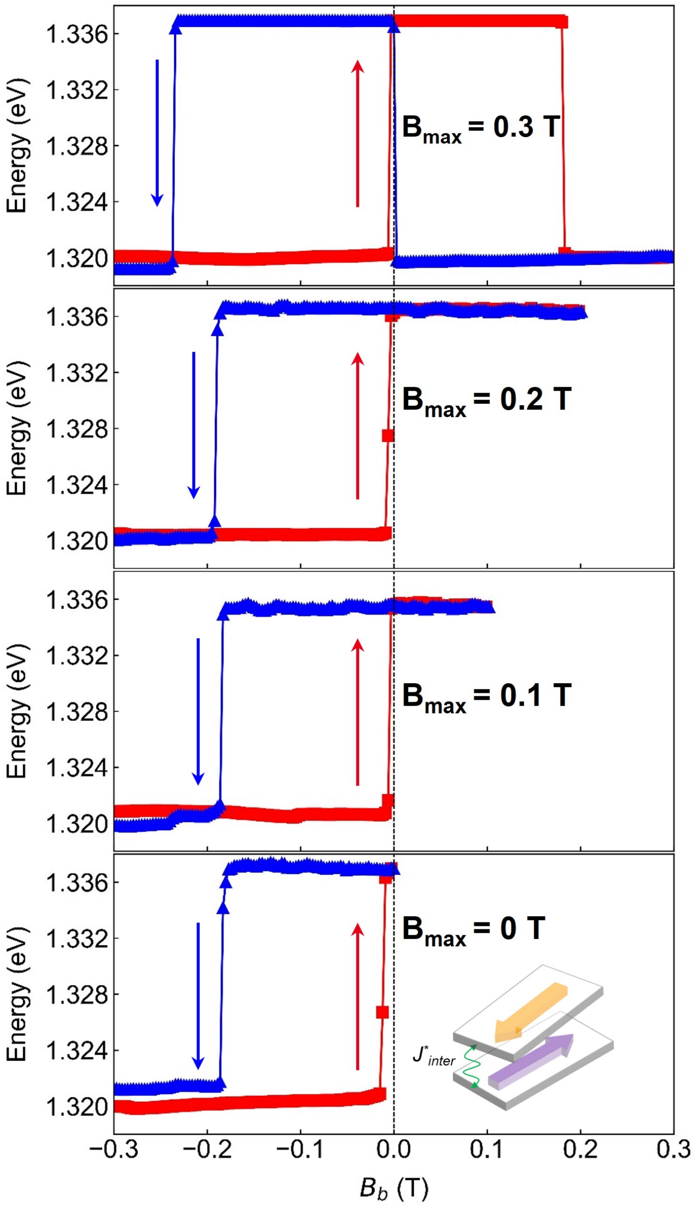

FIG. S10. **Evolution of the magnetic hysteresis curve for the A-exciton with increasing maximum magnetic field ($B_{\mathbf{max}}$) along the b-axis.** The hysteresis curves are displayed vertically in sequential order, starting from the bottom and moving upward, corresponding to maximum fields of $B_{\max}$ = 0T, 0.1T, 0.2T, and 0.3T, respectively. In each case, the magnetic field sweep begins at –0.3T, increases up to the designated $B_{\max}$, and then returns back to –0.3T, completing a closed field cycle.

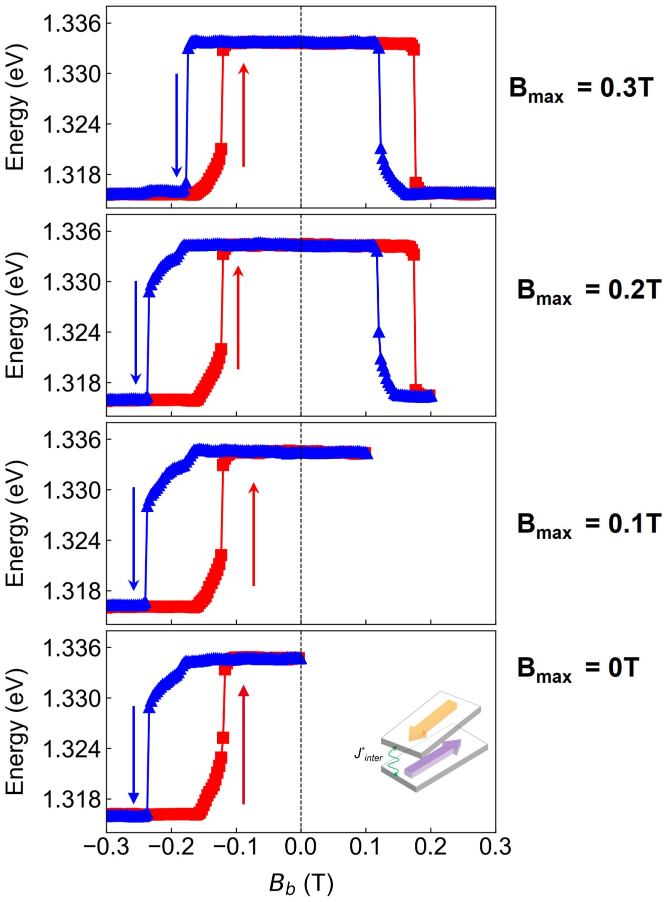

FIG. S11. **Evolution of the magnetic hysteresis curve for the A-exciton with increasing maximum magnetic field ($B_{\mathbf{max}}$) along the b-axis at Spot 3 from the main manuscript figure 4.** The hysteresis curves are displayed vertically in sequential order, starting from the bottom and moving upward, corresponding to maximum fields of $B_{\max}$ = 0T, 0.1T, 0.2T, and 0.3T, respectively. In each case, the magnetic field sweep begins at –0.3T, increases up to the designated $B_{\max}$, and then returns back to –0.3T, completing a closed field cycle.

S7. SUPPLEMENTARY NOTE 7: HYSTERESIS AT DIFFERENT SAMPLE POSITIONS.

Supplementary Note S7 presents the magneto-optical response of the twisted bilayer CrSBr discussed in the main text at two distinct locations, spot 2 and spot 3, focusing on the A-exciton. For each spot, the measurements were performed under magnetic fields applied along all the crystallographic directions, highlighting the anisotropic magnetic behavior. The figures show the exciton photoluminescence (PL) response as the magnetic field is swept in both directions. At both spots, a pronounced magnetic hysteresis is observed along the b-axis, while the a- and c-axis directions show no hysteresis. These results demonstrate that the A-exciton PL is highly sensitive to the local magnetic configuration, and that the hysteretic behavior is robust across different regions of the twisted bilayer.

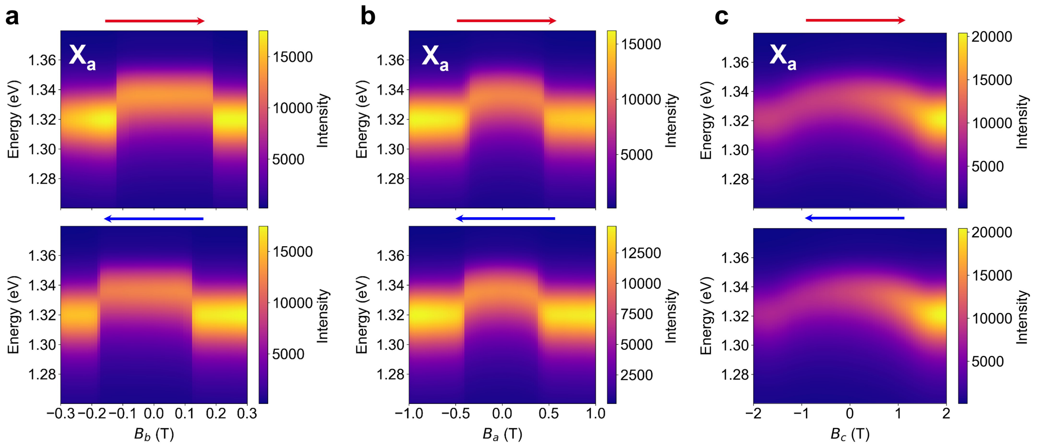

FIG. S12. **Magneto-optical response of the twisted bilayer at spot 2 for the A-exciton, measured under magnetic field applied along specific crystallographic directions. a** shows the magneto-photoluminescence (PL) with the field applied along the b-direction, **b** shows the response for the field along the a-direction, and **c** corresponds to the c-direction. In each case, the top row for each fig shows data for increasing magnetic field (negative to positive), while the bottom row shows the response for decreasing field (positive to negative). A clear magnetic hysteresis is observed when the field is applied along the b-direction, whereas no significant hysteresis is seen for fields applied along the a or c directions.

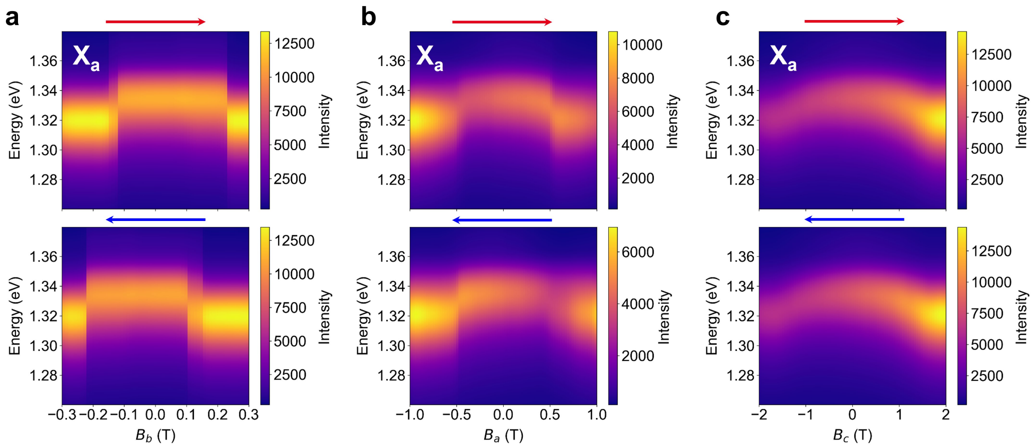

FIG. S13. **Magneto-optical response of the twisted bilayer at spot 3 for the A-exciton, measured under magnetic field applied along three crystallographic directions.a** shows the sample image with the laser spot position for the measurements in dotted circle. **b** shows the magneto-photoluminescence (PL) with the field applied along the b-direction and **c** corresponds to the c-direction. In each case, the top row for each fig shows data for increasing magnetic field (negative to positive), while the bottom row shows the response for decreasing field (positive to negative). A clear magnetic hysteresis is observed when the field is applied along the b-direction, whereas no significant hysteresis is seen for fields applied along the c directions.

S8. SUPPLEMENTARY NOTE 8: MAGNETIC HYSTERESIS IN A ∼2° TWISTED BILAYER–BILAYER DEVICE

To further examine the twist dependence and reproducibility of the observed magnetic hysteresis, we investigate an additional device consisting of a ∼2° twisted bilayer–bilayer structure. Supplementary Fig.S8 presents the magneto-optical response of both a pristine four-layer (4L) sample and the twisted bilayer–bilayer device, measured along the crystallographic *b*-axis.The layer thickness is confirmed by atomic force microscopy.

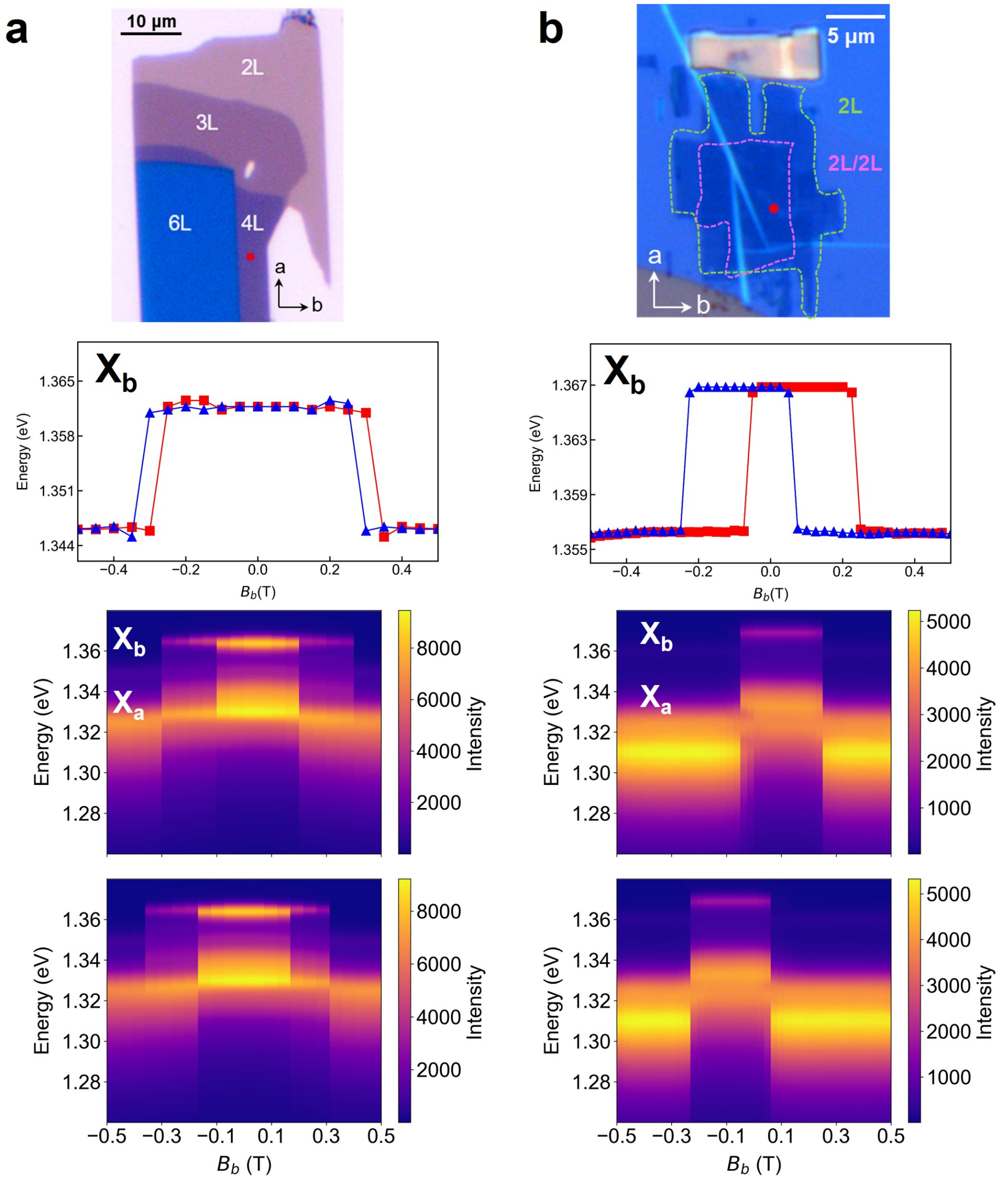

FIG. S14. **Magneto-optical response of pristine 4L and 2° twisted bilayer–bilayer samples along the crystallographic b-axis.** **a** Pristine four-layer (4L) sample **b** 2° twisted bilayer–bilayer sample. In each panel, the top row shows the optical image of the device, with the laser spot indicated by a red circle. The middle row presents the evolution of the B-exciton under an applied magnetic field along the b-axis, highlighting the emergence of magnetic hysteresis in the twisted sample. The bottom row displays the corresponding magnetic field–dependent heatmaps for the pristine and twisted samples. A pronounced hysteresis is observed in the twisted structure, whereas the pristine 4L sample shows no significant hysteresis.

[1] Song, T. *et al.* Direct visualization of magnetic domains and moiré magnetism in twisted 2D magnets. *Science* **374**, 1140–1144 (2021).

[2] Xu, Y. *et al.* Coexisting ferromagnetic–antiferromagnetic state in twisted bilayer CrI_3. *Nature Nanotechnology* **17**, 143–147 (2022).

[3] Cheng, G. *et al.* Electrically tunable moiré magnetism in twisted double bilayers of chromium triiodide. *Nature Electronics* **6**, 434–442 (2023).

[4] Scheie, A. *et al.* Spin Waves and Magnetic Exchange Hamiltonian in CrSBr. *Advanced Science* **9**, 2202467 (2022).

[5] Ziebel, M. E. *et al.* CrSBr: An Air-Stable, Two-Dimensional Magnetic Semiconductor. *Nano Letters* **24**, 4319 (2024).

[6] Dirnberger, F. *et al.* Magneto-optics in a van der waals magnet tuned by self-hybridized polaritons. *Nature* **620**, 533–537 (2023).

[7] Cham, T. M. J. *et al.* Anisotropic gigahertz antiferromagnetic resonances of the easy-axis van der Waals antiferromagnet CrSBr. *Nano Letters* **22**, 6716–6723 (2022).

[8] Gurevich, A. & Melkov, G. *Magnetization Oscillations and Waves* (Taylor & Francis, 1996). URL https://books.google.de/books?id=YgQtSvFIvFQC.

[9] Li, H.-F. Possible ground states and parallel magnetic-field-driven phase transitions of collinear antiferromagnets. *npj Computational Materials* **2**, 16032 (2016).

[10] Stewart, J. *Multivariable Calculus: Concepts and Contexts* (Thomson Brooks/Cole, 2005). URL https://books.google.de/books?id=eNHhKxXCJaEC.

[11] Xu, Y. *et al.* Coexisting ferromagnetic–antiferromagnetic state in twisted bilayer cri3. *Nature Nanotechnology* **17**, 143–147 (2022). URL https://doi.org/10.1038/s41565-021-01014-y.

[12] Song, T. *et al.* Giant tunneling magnetoresistance in spin-filter van der waals heterostructures. *Science* **360**, 1214–1218 (2018). URL https://www.science.org/doi/abs/10.1126/science.aar4851. https://www.science.org/doi/pdf/10.1126/science.aar4851.

[13] Sivadas, N., Okamoto, S., Xu, X., Fennie, C. J. & Xiao, D. Stacking-dependent magnetism in bilayer cri3. *Nano Letters* **18**, 7658–7664 (2018). URL https://doi.org/10.1021/acs.nanolett.8b03321.

[14] Yang, K., Wang, G., Liu, L., Lu, D. & Wu, H. Triaxial magnetic anisotropy in the two-

dimensional ferromagnetic semiconductor crsbr. *Phys. Rev. B* **104**, 144416 (2021). URL https://link.aps.org/doi/10.1103/PhysRevB.104.144416.

[15] Cham, T. M. J. *et al.* Anisotropic gigahertz antiferromagnetic resonances of the easy-axis van der waals antiferromagnet crsbr. *Nano Letters* **22**, 6716–6723 (2022). URL https://doi.org/10.1021/acs.nanolett.2c02124.

[16] Wilson, N. P. *et al.* Interlayer electronic coupling on demand in a 2D magnetic semiconductor. *Nature Materials* **20**, 1657–1662 (2021).

[17] Tabataba-Vakili, F. *et al.* Doping-control of excitons and magnetism in few-layer CrSBr. *Nature Communications* **15**, 4735 (2024).

[18] Krelle, L. *et al.* Magnetic correlation spectroscopy in CrSBr. *ACS Nano* **19**, 33156–33163 (2025).

[19] Shree, S., Paradisanos, I., Marie, X., Robert, C. & Urbaszek, B. Guide to optical spectroscopy of layered semiconductors. *Nature Reviews Physics* **3**, 39–54 (2021).
