# Supplementary material for: Twist-tuned exchange and hysteresis in a bilayer van der Waals magnet
Source: Nat Commun. 2026 Jul 8;17:5984. doi: 10.1038/s41467-026-75186-3 (PMC13346542; doi:10.1038/s41467-026-75186-3)
Supplement: Supplementary file 2 — Transparent Peer Review file [file 41467_2026_75186_MOESM2_ESM.pdf]

nature portfolio

Peer Review File

Twist-tuned exchange and hysteresis in a bilayer van der Waals magnet

Corresponding Author: Professor Bernhard Urbaszek

This file contains all reviewer reports in order by version, followed by all author rebuttals in order by version.

Version 0:

Reviewer comments:

Reviewer #1

(Remarks to the Author)
The work by Mondal et al. reports on magneto-optical experiments (photoluminescence under applied magnetic fields) on a twisted magnetic bilayer of CrSBr. CrSBr is a van der Waals magnet that has recently gained a lot of attention due to its magnetic, optical, and electrical properties from bulk down to the single layer, being the topic of interest for a broad community in condensed matter physics.

By twisting 3 degrees, the authors observe the appearance of magnetic hysteresis (which is absent in the pristine bilayer). Interestingly, this hysteresis exhibits a spatial dependence. The authors interpret these results as a twist-induced tunable (effective) interlayer exchange coupling, based on their theoretical model.
The theoretical model develops the free energy of two-magnetic sublattices. However, within this approach, all observed effects are effectively attributed to the (effective) interlayer exchange constant (the authors do not consider changes in magnetic anisotropy and, for CrSBr, the interlayer ferromagnetic exchange is ferromagnetic). Thus, this assumption overlooks other relevant contributions, as local strain (which can yield to the formation of magnetic domains). Indeed, some of the hysteresis features in the current study are not captured by the model, showing that strain or other local inhomogeneities play a significant role. For example, local strains -that are unavoidable while working with mechanically transferred flakes- are reported to yield to hysteretic and non-homogeneous magnetic switching in twisted-CrSBr (ACS Nano, doi: 10.1021/acsnano.5c12297).

Regarding the novelty of the work, I have some concerns. Although it is the first report on the magnetic hysteresis in twisted-CrSBr probed by photoluminescence experiments, the results could be expected since the emergence of hysteresis in twisted-CrSBr bilayers is known by magneto-transport and magnetic imaging measurements (Nature 2024, 632, 1045; Nature Materials 2024, 23, 212; Advanced Materials 2025, 37, 8, 2415774; ACS Nano 2025, DOI: 10.1021/acsnano.5c12297) and CrSBr exhibits a strong coupling between the magnetic order and the optical properties (Nature Materials 2021, 20, 1657). Theoretically, the twist-tuned exchange effects in CrSBr are discussed as well based on first-principle calculations (PNAS 2024, DOI: 10.1073/pnas.2413326121; J. Phys. Chem. C 2025, 129, 37, 16897–16910) and the current developed model exhibits some limitations while reproducing the experiments.

Overall, I find that other effects (as strain), and not simply the exchange, could be behind the experimental observations (in particular, in the spatial mapping). Therefore, I cannot recommend its publication in Nature Communications at the present stage.

In particular:

1.- The authors discuss the effect of the moiré superlattice on the interlayer exchange coupling (A or M points in Figure 1, “The interlayer exchange is expected to be position dependent following a moiré superlattice,” page 1). However, the measurements are performed optically with a spot size of approximately 500 nm (I may have missed it but, which one is the size of the optical spot used in the measurements?). Given that the moiré period is smaller than this, do the authors have sufficient spatial resolution to resolve moiré effects and, therefore, the local variation of the interlayer exchange constant depending on the AA/AB stacking? Otherwise, which one is the physical origin of the effective interlayer exchange constant discussed in the model?

2.- The observed spatial variation in the field-sweep reversals is interesting, but how can the authors rule out other origins, as local strain? Mechanically transferred flakes inevitably feature strain inhomogeneities, which are known to influence magnetic switching in twisted-CrSBr (ACS Nano 2025, DOI: 10.1021/acsnano.5c12297). Can the authors provide a local strain map to support the attribution of these effects to a twist-induced reduction of the interlayer exchange (and discard local strain effects)? The distance between the measured positions is large and, therefore, do not suggest any correlation with the underlying Moiré (unless the Moiré is not homogeneous).

3.- The manuscript presents results from a single device. How reproducible are these effects? It would strengthen the work to include data from at least a second twisted bilayer device or show how the twist-angle correlates with the calculated (effective) interlayer exchange constant.

4.- The authors state that the system exhibits “an effective monodomain response characterized by switching into the antiferromagnetic state, rather than forming spin textures or fragmented domains.” However, in Fig. 4d (position 3), the hysteresis curve clearly shows two-step switching. Such features have previously been attributed to domain formation (Advanced Materials 2025, 37, 8, 2415774; ACS Nano 2025, DOI: 10.1021/acsnano.5c12297), likely associated with strain inhomogeneities. To verify the monodomain interpretation, can the authors perform minor-loop measurements at position 3, analogous to those shown for position 1 in Fig. S10?

Reviewer #2

(Remarks to the Author)
Since the first studies on twisted CrSBr (such as Parkin group’s Nature) focused on relatively large twist angles, I’m curious how is the magnetism for small twist angles-whether it shows the coexistence of FM-AFM phases as in CrI3 case, or instead maintains collinear configurations. Here the PL experiments by P. Mondal et al. appear to support the latter. Another interesting point, from the perspective of application, the magnetic transitions are observed close to zero field. The manuscript is well written with clear experimental evidence, and I believe its novelty is sufficient to meet the standards of Nat. Commun. I would recommend publication provided the following concerns are well addressed:
(1) One concern is the assumed two-sublattice model. The authors believe the spins in each layer are uniform and aligned in the same direction, due to the strong intralayer exchange in CrSBr. This might be reasonable if the moiré period is very small and the domain wall (DW) formation is too energetically costly. However, instead of sharp 180 or 90 degree DWs, one could also imagine a spatially-varying spin texture within one layer, with small angle deviations, which should be a noncollinear structure, strictly speaking. The authors mentioned that the obtained critical field is not adequately captured by the theory. Is this discrepancy relevant to the possible noncollinear texture ? Do Fig. 4c,d support this scenario ?
(2) The directions of the ground state spins (M_A and M_B) are not clearly specified. Do they align with the easy b axes of their respective layer, or along a common direction between the two easy axes?
(3) The authors mention “twist-tuned lowering of the interlayer antiferromagnetic exchange”, why must the exchange be lowering? Are there any theoretical calculations supporting this?
(4) What is the precise meaning of coherence in the context of twisted magnets ?
(5) In intrinsic 2D magnets, multiple domains have been reported (such as doi.org/10.1038/s41467-021-22239-4). What is the role of those multiple domains in your system ?
(6) In a generic bilayer AFM, when the field is applied along the easy axis, the critical field is determined by the interlayer exchange, while the hysteresis between upward and downward sweeps is determined by anisotropy K. Does the pronounced hysteresis imply that K is much larger in twisted CrSBr?
(7) If the field is not applied along easy axis, the critical field is determined by exchange plus anisotropy. Why the critical field for B//c is larger in the twisted case (comparing Fig. 2b,d)? Does it mean the exchange is enhanced ?

Version 1:

Reviewer comments:

Reviewer #1

(Remarks to the Author)
I thank the authors for addressing my comments.

I raise two main points and one minor comment (see below). Based on the authors’ response, I believe our understanding of the underlying process is aligned. I hope these comments help clarify the message for a broader audience.

Overall, I am happy to recommend publication in Nature Communications once the authors address and clarify these points.

1. As a first point, in their response the authors argue that: (1) their model, in the absence of strain due to experimental constraints, is expected to exhibit a spatially homogeneous magnetic state, switching, and hysteresis; and (2) their spatial mapping (which shows local modifications) highlights the importance of strain and lattice reconstruction due to twisting, and is therefore consistent with the reviewer’s comment.

The inhomogeneities in the experimental samples are not a critical issue (in my view), since such variations in local strain are unavoidable when working with mechanically exfoliated and transferred 2D materials. Beyond moiré reconstruction, the formation of wrinkles and local strain variations during the transfer process is, to some extent, inevitable.

However, my main concern is that the authors develop a model (which is perfectly valid), but this model is not directly applicable to their experimental dataset, where local variations and additional effects such as strain play an important role. Despite this, the authors use the experimental data to extract parameters based on their model. Therefore, these extracted values should be interpreted with caution.

It would be useful for the authors to clearly acknowledge and discuss this point in the manuscript.

2. As a second point, the authors claim that the layers behave as a monodomain (page 5. “Our twisted bilayer sample seems to manifest sharp monodomain Stoner-Wohlfarth switching and does not offer any evidence of domains”). However, based on the data in Fig. 4, at a field of, for example, –0.1 T (red curve), position 1 is in a different state compared to positions 2 and 3. Thus, it is not clear how the switching can be described as monodomain for the whole wisted bilayer sample. At single locations the system may be described as such (in particular, for position 1 with a very sharp transition), but not for the whole twisted heterostructure (in position 2 and 3 there are two processes ocurring: a sharp energy change and a more gradual one).

In addition, the field values used in Fig. S11 to support the monodomain claim are not well chosen. A minor loop would likely appear in Fig. S11 if a maximum field B_max of, for example, –0.15 T were applied, but not for fields > 0 T. Regarding Fig. S10, I agree that the switching appears consistent with a monodomain behavior for that location.

Thus, the experimental data do not seem to support a monodomain scenario across the entire twisted bilayer. Therefore, I do not find the claim of monodomain switching across all positions to be fully supported. That said, I do not consider this a critical issue for the manuscript; softening or removing this claim would not affect the main conclusions. However, from data show in Fig 4, it is experimentally clear that different spin-switching processes are taken place considering the whole twisted-bilayer, incompatible with a monodomain switch across the whole heterostructure.

Minor comment: I note that references 19 and 36 (Boix-Constant et al.) are duplicates, as are references 32 and 47 (Liu et al.).

Reviewer #2

(Remarks to the Author)
I appreciate the authors' responses addressing my precious comments.
However, the most critical point, which was raised by Reviewer#1, is that all data for the twisted crsbr were acquired from only a SINGLE device with 3degree twist. This raises severe concerns that the observed hysteresis highlighted in the main claim may just originate from extrinsic factors like strain or stacking faults, rather than moire-modulated exchange/anisotropy. Without angle dependent experiments and reproducible devices, their claim would remains unsubstantiated.
Therefore, I cannot recommend its publication until such supportive experimental evidence is provided.

Version 2:

Reviewer comments:

Reviewer #2

(Remarks to the Author)
The authors have addressed all my concerns, and I'm happy to recommend the manuscipt for publication in Nat. Commun.

Open Access This Peer Review File is licensed under a Creative Commons Attribution 4.0 International License, which permits use, sharing, adaptation, distribution and reproduction in any medium or format, as long as you give appropriate credit to the original author(s) and the source, provide a link to the Creative Commons license, and indicate if changes were made.
In cases where reviewers are anonymous, credit should be given to 'Anonymous Referee' and the source.
The images or other third party material in this Peer Review File are included in the article's Creative Commons license, unless indicated otherwise in a credit line to the material. If material is not included in the article's Creative Commons license and your intended use is not permitted by statutory regulation or exceeds the permitted use, you will need to obtain permission directly from the copyright holder.
To view a copy of this license, visit https://creativecommons.org/licenses/by/4.0/

Point-by-Point Response to the Referees

We sincerely thank the referees for the time and effort they dedicated to reviewing our manuscript and for their valuable comments, which have greatly improved our work. All points raised have been carefully addressed and discussed below, along with the corresponding modifications made to the text.

Reviewer #1 (Remarks to the Author):

Reviewer #1 General Comments

The work by Mondal et al. reports on magneto-optical experiments (photoluminescence under applied magnetic fields) on a twisted magnetic bilayer of CrSBr. CrSBr is a van der Waals magnet that has recently gained a lot of attention due to its magnetic, optical, and electrical properties from bulk down to the single layer, being the topic of interest for a broad community in condensed matter physics.

Response

We thank the reviewer for summarizing our work and for noting its high relevance for a broad scientific community.

Reviewer #1 General Comments

By twisting 3 degrees, the authors observe the appearance of magnetic hysteresis (which is absent in the pristine bilayer). Interestingly, this hysteresis exhibits a spatial dependence. The authors interpret these results as a twist-induced tunable (effective) interlayer exchange coupling, based on their theoretical model. The theoretical model develops the free energy of two-magnetic sublattices. However, within this approach, all observed effects are effectively attributed to the (effective) interlayer exchange constant (the authors do not consider changes in magnetic anisotropy and, for CrSBr, the interlayer ferromagnetic exchange is ferromagnetic).

Response

There seems to have been a misunderstanding here. Please allow us to clarify.

Our theoretical model and the corresponding interpretation of experiments duly account for changes in the magnetic anisotropy. This has been discussed in the main text and detailed in Supplementary Note 2, where changes in anisotropy have been treated on an equal footing with the interlayer exchange.

Changes in manuscript

In the revised manuscript, we now further clarify this in the caption of Fig. 1, for example. While modification of the interlayer exchange with the twist-angle is rather intuitive, a change in magnetic anisotopy is also allowed and accounted for by our theoretical analysis.

Reviewer #1 General Comments

Thus, this assumption overlooks other relevant contributions, as local strain (which can yield to the formation of magnetic domains). Indeed, some of the hysteresis features in the current study are not captured by the model, showing that strain or other local inhomogeneities play a significant role. For example, local strains -that are unavoidable while working with mechanically transferred flakes- are reported to yield to hysteretic and non-homogeneous magnetic switching in twisted-CrSBr (ACS Nano, doi: 10.1021/acsnano.5c12297).

Response

This comment by the reviewer also seems to be rooted in the misunderstanding mentioned above. Please allow us to clarify.

As per our theoretical analysis, a strain-free twisted bilayer with our sample's twist angle of $\sim 3^\circ$ would manifest a complete spatial homogeneity in the switching patterns. Hence, our spatial mapping of the hysteresis and resulting extraction of spatially-resolved interlayer exchange and anisotropy values (Supplementary Note 2) directly addresses the importance of the strain and lattice reconstruction due to twisting, and is consistent with the reviewer's comment.

A key message of our work is that spatial inhomogeneity and strain are not necessary for engineering the hysteresis and switching behavior in these magnets. This is in sharp contrast with the picture presented in some of the previous literature and offers a significant step forward. A similar message employing a Landau-Lifshitz theoretical description similar to ours is delivered by the publication: *Ferromagnet-like binary switching of a Stoner–Wohlfarth antiferromagnet*, Nature 650, 340 (2026), which has been published recently, well after the submission of our manuscript. **Hence, we request the reviewer and editor to process our manuscript rapidly.**

The reference mentioned by the reviewer [*Imaging Magnetic Switching in Orthogonally Twisted Stacks*

of a van der Waals Antiferromagnet, ACS Nano 19, 42140 (2025)] discusses spatially inhomogeneous switching behavior in *orthogonally-twisted* CrSBr bilayers. They attribute the spatial inhomogeneity to strain, fully consistent with our approach. However, the nature of magnetic switching and hysteresis in such *large-angle twisted* samples is fundamentally different from our *small-angle twisted* sample. This difference will be detailed in a separate theoretical work.

Changes in manuscript

In the revised manuscript,

- we provide an additional detailed calculation in Supplementary Note 3 which demonstrates that our twisted bilayer sample, in the absence of strain due to experimental constraints, is expected to manifest spatially homogeneous magnetic state, switching and hysteresis.
- we further emphasize the qualitative difference between our *small-angle twisted* sample and an *orthogonally-twisted* sample.

Reviewer #1 General Comments

Regarding the novelty of the work, I have some concerns. Although it is the first report on the magnetic hysteresis in twisted-CrSBr probed by photoluminescence experiments, the results could be expected since the emergence of hysteresis in twisted-CrSBr bilayers is known by magneto-transport and magnetic imaging measurements (Nature 2024, 632, 1045; Nature Materials 2024, 23, 212; Advanced Materials 2025, 37, 8, 2415774; ACS Nano 2025, DOI: 10.1021/acsnano.5c12297) and CrSBr exhibits a strong coupling between the magnetic order and the optical properties (Nature Materials 2021, 20, 1657). Theoretically, the twist-tuned exchange effects in CrSBr are discussed as well based on first-principle calculations (PNAS 2024, DOI: 10.1073/pnas.2413326121; J. Phys. Chem. C 2025, 129, 37, 16897–16910) and the current developed model exhibits some limitations while reproducing the experiments.

Response

To adequately comment on the novelty of our work, let us first list each of the articles mentioned by the reviewer:

1. *Multistep magnetization switching in orthogonally twisted ferromagnetic monolayers*, Nature Materials 23, 212 (2024). (already cited in our previous submission)

   This article experimentally (supported by micromagnetic simulations) addresses a CrSBr bilayer

with the large 90° twist angle ("orthogonally twisted"), which does NOT result in a moiré pattern.

2. *Twist-assisted all-antiferromagnetic tunnel junction in the atomic limit*, Nature 632, 1045 (2024). (already cited in our previous submission)
   This article experimentally studies electronic transport through *large-angle twisted* CrSBr layers. The non-zero twist angles are 10° or larger. Further, spatial resolution is not afforded by the conducted electrical measurements.

3. *Moiré magnetism and moiré excitons in twisted CrSBr bilayers*, Proc. Natl. Acad. Sci. 122, e2413326121 (2025). (already cited in our previous submission)
   This article presents a first-principles numerical calculation of magnetic exchange and anisotropy as well as electronic bandstructure parameters in a small to large-angle twisted CrSBr bilayers. It does not discuss any evolution of the magnetic states with applied magnetic fields or the consequent hysteresis.

4. *Programmable Magnetic Hysteresis in Orthogonally-Twisted 2D CrSBr Magnets via Stacking Engineering*, Adv. Mater. 37, 2415774 (2025). (already cited in our previous submission)
   This article experimentally (supported by micromagnetic simulations) addresses switching in *orthogonally-twisted* CrSBr multilayers. No moiré pattern expected.

5. *Imaging Magnetic Switching in Orthogonally Twisted Stacks of a van der Waals Antiferromagnet*, ACS Nano 19, 42140 (2025). (cited in the revised manuscript)
   This pertains to experiments on strain-mediated spatially inhomogeneous magnetic states and switching in *orthogonally-twisted* bilayers of CrSBr.

6. *Twist-Engineered Switching of Magnetic Phase and Anisotropy in Bilayer CrSBr: A First-Principles Study*, J. Phys. Chem. C 129, 16897 (2025). (cited in the revised manuscript)
   Very similar to article no. 3 listed above: a numerical evaluation of exchange and anisotropy parameters in twisted CrSBr bilayers.

Now, the novelty of our work becomes evident on recognizing the following two central points:

- **The qualitative difference between our *small-angle* twisted bilayers and above referenced *large-angle* (including orthogonally) twisted bilayers cannot be overemphasized**. This is well understood from the field of twisted bilayer graphene and transition metal dichalcogenides. For example, see *The marvels of moiré materials*, Nature Reviews Materials 6, 201 (2021). Please

consider that all the exciting phenomena here, as well as experimental challenges due to lattice reconstruction, are found for small-angle twisted samples only. Our work addresses such a coherent effect of small-twist angle moiré superlattice uncovering, for the first time, switching and hysteresis in a monodomain small-twist magnetic bilayer.

- **Our Landau-Lifshitz description based analytic framework provides a paradigm shift in how the magnetic state evolution is understood in this field** (similar to, and a bit more powerful than, the paradigm shift contributed by the recent publication: Nature 650, 340 (2026), which has appeared after our submission). Only because our results are analytic, we can extract the exchange and anisotropy parameters from the measured hysteresis. Please consider that the entire field of magnetism including its switching and dynamical excitations is built on such Landau-Lifshitz descriptions. While first-principle calculations are useful for theoretically estimating magnetic interactions, they simply provide parameters for use in Landau-Lifshitz descriptions. Our analysis allows one to obtain these parameters directly from switching experiments.

Changes in manuscript

We now clarify this novelty and contrast with large-twist samples more emphatically in our revised manuscript.

Reviewer #1 General Comments

Overall, I find that other effects (as strain), and not simply the exchange, could be behind the experimental observations (in particular, in the spatial mapping). Therefore, I cannot recommend its publication in Nature Communications at the present stage.

Response

As per the discussion above, this assessment by the reviewer appears to be rooted in a misunderstanding of our manuscript combined with a lack of emphasis on the fundamental difference between *large-angle* and *small-angle* twisted bilayers. Our revised manuscript clarifies these misunderstandings. Thus, we firmly believe that our work is now ready for publication in Nature Communications.

Reviewer #1 Specific Comment 1

In particular:

1.- The authors discuss the effect of the moiré superlattice on the interlayer exchange coupling (A or M points in Figure 1, "The interlayer exchange is expected to be position dependent following a moiré superlattice," page 1). However, the measurements are performed optically with a spot size of approximately 500 nm (I may have missed it but, which one is the size of the optical spot used in the measurements?). Given that the moiré period is smaller than this, do the authors have sufficient spatial resolution to resolve moiré effects and, therefore, the local variation of the interlayer exchange constant depending on the AA/AB stacking? Otherwise, which one is the physical origin of the effective interlayer exchange constant discussed in the model?

Response

We thank the reviewer for pointing out this source of potential confusion.

Indeed, our moiré wavelength, estimated to be around 10nm, is significantly smaller than both the optical spot size and the intralayer exchange length. Thus, the excitons as well as PL sample a spatial average (over the moiré pattern) of the magnetic interlayer exchange and anisotropy parameters. Here, the emphasis has to be on the intralayer ferromagnetic exchange length, which is relatively large in CrSBr. It is this determining factor that enforces an effective monodomain behavior in this material, as compared to CrI_3, for example.

If our samples manifested the ideal moiré pattern (without any strain-related variations) with this moiré wavelength of about 10nm, there would be no spatial dependence in our PL measurements.

Changes in manuscript

We have now added clarifications around the statement quoted by the referee. We also highlight the key role of the intralayer ferromagnetic exchange length in enforcing a monodomain magnetic behavior in our sample. This has been further detailed in the new Supplementary Note 3 via additional theoretical analysis.

Reviewer #1 Specific Comment 2.1

2.- The observed spatial variation in the field-sweep reversals is interesting, but how can the authors rule out

other origins, as local strain? Mechanically transferred flakes inevitably feature strain inhomogeneities, which are known to influence magnetic switching in twisted-CrSBr (ACS Nano 2025, DOI: 10.1021/acsnano.5c12297). Can the authors provide a local strain map to support the attribution of these effects to a twist-induced reduction of the interlayer exchange (and discard local strain effects)?

Response

Sorry for this misunderstanding. Indeed, our observed spatial variation is entirely attributed to the strain (or lattice reconstruction). Thus, we may treat our ascertained spatially-resolved values of interlayer exchange and magnetic anisotropy as a strain map.

Our attribution of a change in the magnetic parameters to twist-tuning relies on our observation of ideal switching, consistent with our monodomain theoretical framework, in regions with the linear size of about 500 nm. It is expected that the twist-angle and the consequent moiré pattern in our sample is somewhat inhomogeneous, as is common in small-twist samples, due to strain and lattice reconstruction.

Changes in manuscript

We now clarify the role of strain or lattice reconstruction in causing spatial variations in our sample.

Reviewer #1 Specific Comment 2.2

The distance between the measured positions is large and, therefore, do not suggest any correlation with the underlying Moiré (unless the Moiré is not homogeneous).

Response

Agreed. Please see the response above. The spatial variation is due to strain and lattice reconstruction.

Reviewer #1 Specific Comment 3

3.- The manuscript presents results from a single device. How reproducible are these effects? It would strengthen the work to include data from at least a second twisted bilayer device or show how the twist-angle correlates with the calculated (effective) interlayer exchange constant.

Response

Our theoretical and experimental claim is that our bilayer sample manifests spatially homogeneous Stoner-Wohlfarth switching over a region few 100 nm size. Thus, demonstrating this behavior at several different regions addresses the reproducibility of the phenomenon. For conventional crystalline magnets, this would be analogous to cutting a sample into two and calling it two different devices for reproducibility.

Unfortunately, due to the widely-recognized difficulties in realizing bilayers with small twists and our intercontinental collaboration on samples, we were not able to achieve completely new samples within the time constraints of this reply.

Changes in manuscript

We have now added Supplementary Note 3 providing detailed theoretical analysis explaining the expected spatial homogeneity as well as critical twist angles in our CrSBr sample, if there were no strain or lattice reconstruction.

Reviewer #1 Specific Comment 4

4.- The authors state that the system exhibits "an effective monodomain response characterized by switching into the antiferromagnetic state, rather than forming spin textures or fragmented domains." However, in Fig. 4d (position 3), the hysteresis curve clearly shows two-step switching. Such features have previously been attributed to domain formation (Advanced Materials 2025, 37, 8, 2415774; ACS Nano 2025, DOI: 10.1021/acsnano.5c12297), likely associated with strain inhomogeneities. To verify the monodomain interpretation, can the authors perform minor-loop measurements at position 3, analogous to those shown for position 1 in Fig. S10?

Response

Thanks for highlighting this source of confusion.

We have now carried out the suggested additional measurements and found them to be fully consistent with our understanding in terms of monodomain switching.

As noted above, our theoretical and experimental analysis are in full consistence with a monodomain

model thereby highlighting the Stoner-Wohlfarth nature of the observed switching. Further, as also discussed above, our results on a small-twist sample are not directly comparable with the orthogonally twisted samples discussed in the reference mentioned by the referee.

Changes in manuscript

We carried out the measurements as suggested by the reviewer and they are now reported in Fig. S11 (Supplementary Note 6) and are found to support our interpretation in terms of monodomain switching behavior. Furthermore, our additional theoretical analysis discussed now in Supplementary Note 3 further justifies that our sample with a twist angle of 3° is expected to manifest a monodomain behavior.

Reviewer #2 (Remarks to the Author):

Reviewer #2 General Comments

Since the first studies on twisted CrSBr (such as Parkin group's Nature) focused on relatively large twist angles, I'm curious how is the magnetism for small twist angles-whether it shows the coexistence of FM-AFM phases as in CrI3 case, or instead maintains collinear configurations. Here the PL experiments by P. Mondal et al. appear to support the latter. Another interesting point, from the perspective of application, the magnetic transitions are observed close to zero field. The manuscript is well written with clear experimental evidence, and I believe its novelty is sufficient to meet the standards of Nat. Commun. I would recommend publication provided the following concerns are well addressed:

Response

We thank the reviewer for their evaluation of our manuscript, recognizing the novelty of our study, and constructive feedback.

We address the reviewer's specific comments below.

Reviewer #2 Specific Comment 1

(1) One concern is the assumed two-sublattice model. The authors believe the spins in each layer are uniform and aligned in the same direction, due to the strong intralayer exchange in CrSBr. This might be reasonable if the moiré period is very small and the domain wall (DW) formation is too energetically costly. However, instead of sharp 180 or 90 degree DWs, one could also imagine a spatially-varying spin texture within one layer, with small angle deviations, which should be a noncollinear structure, strictly speaking. The authors mentioned that the obtained critical field is not adequately captured by the theory. Is this discrepancy relevant to the possible noncollinear texture ? Do Fig. 4c,d support this scenario ?

Response

We thank the referee for this insightful comment.

The lengthscale of a slowly varying spin texture within one layer, such as domain wall, is indeed governed by the intralayer exchange. This length increases with the strength of the intralayer ferromagnetic exchnage and when it exceeds the moiré wavelength, the spin texture becomes energetically less favorable than the spatially homogeneous state. In our case, due to the large intralayer

ferromagnetic exchange, we find this spin texture lengthscale to be larger than our moiré wavelength, thereby finding the spatially homogeneous magnetic state to be relevant. The same conclusion is reached via energy comparisons, instead of the lengttscale.

Hence, we do not see our data to offer any support in favor of spin texture scenario. At the same time, due to the inevitable strains and spatial variation over micrometer lengthscales in small-angle twisted sample, an unpredictable spin texture cannot be fully ruled out.

Our critical field is adequately captured in all, but one, cases. And even in the case where it does not seem to be fully captured, we prefer to not draw strong conclusions since our model does not fully take into account pinning potentials. We expect that based on the present work, we or the scientific community members should be able to systematically address this in the near future.

Changes in manuscript

We now provide additional calculations in the new Supplementary Note 3 comparing the textured and monodomain magnetic states for various twist angles in our CrSBr sample and the previously studied CrI_3. This analysis further supports our inference that we must have monodomain magnetic state in our sample.

Reviewer #2 Specific Comment 2

(2) The directions of the ground state spins (M_A and M_B) are not clearly specified. Do they align with the easy b axes of their respective layer, or along a common direction between the two easy axes?

Response

For the small-angle twisted sample under consideration, the distinction between the local b axes in the two layers turns out to not be important. Indeed, our experiments performed by changing the applied magnetic field direction within these 3° did not distinguish the observations. Consequently, we keep our theoretical model simple and do not explicitly account for this small misalignment.

Changes in manuscript

We now clarify this issue of slight misalignment and its not being important for the phenomena under investigation.

Reviewer #2 Specific Comment 3

(3) The authors mention "twist-tuned lowering of the interlayer antiferromagnetic exchange", why must the exchange be lowering? Are there any theoretical calculations supporting this?

Response

Our assertion about the "lowering" of the interlayer exchange is based on the following two observations:

- From our experiments, we are able to extract the interlayer exchange by comparing the theoretical model with the observed hysteresis. In this, we find a lowering of the exchange.
- From theory perspective, previous first-principles calculations (*Moiré magnetism and moiré excitons in twisted CrSBr bilayers*, Proc. Natl. Acad. Sci. 122, e2413326121 (2025)) suggest that the interlayer exchange becomes ferromagnetic is some parts of the moiré superlattice. Since our monodomain state spatially averages over the entire moiré cell, the ferromagnetic contributions in some parts lead to a reduction of the interlayer exchange which still remains antiferromagnetic overall.

Changes in manuscript

We now further clarify this consistence between theoretical expectations and experimental observation.

Reviewer #2 Specific Comment 4

(4) What is the precise meaning of coherence in the context of twisted magnets ?

Response

To adequately comment on the significance of "coherence", we must compare with the electrons in a moiré lattice. The electronic band structure is formed based on the potentials that electrons can experience within their coherence length. Thus, it is fundamentally important that the moiré wavelength is shorter than the electron coherence length to observe all the beautiful moiré physics in twisted

graphene and TMDs.

In a similar manner, if the magnetization (together with its magnonic excitations) experiences the spatially varying spin interactions as one unit, only then can one expect emergence of moiré bands and physics. If the magnetization breaks into spatial domains, effects related to moiré bandstructure are lost.

Changes in manuscript

We briefly clarify this in the revised manuscript, highlighting the analogy between electrons and magnons.

Reviewer #2 Specific Comment 5

(5) In intrinsic 2D magnets, multiple domains have been reported (such as doi.org/10.1038/s41467-021-22239-4). What is the role of those multiple domains in your system ?

Response

Due to the larger intralayer ferromagnetic exchange interaction in CrSBr, it is better than most van der Waals magnets at avoiding intrinsic spin textures and domains. Comparing our data to other observations in the literature, we do not find a strong evidence of domains.

Since switching may proceed via the formation of domains, our sample is likely to have around the switching field thereby broadening the switching process. This is however quite different from the observation in other van der Waals magnets and is common phenomnology for even the conventional magnets. Furthermore, the spatial inhomogeneity and strain on large lengthscales in our sample should inevitably lead to some domains.

Changes in manuscript

We now discuss the relevance of domains while comparing with and citing the reference that the referee brings to our attention.

Reviewer #2 Specific Comment 6

(6) In a generic bilayer AFM, when the field is applied along the easy axis, the critical field is determined by the interlayer exchange, while the hysteresis between upward and downward sweeps is determined by anisotropy K. Does the pronounced hysteresis imply that K is much larger in twisted CrSBr?

Response

Yes, in a way. Upon twisting, the interlayer exchange becomes weaker which means that the K becomes larger relative to the exchange, thereby widening the hysteresis.

We have rigorously derived and discussed these different fields in Supplementary Note 1 and summarized them in Table S1 and Fig. S2.

Reviewer #2 Specific Comment 7

(7) If the field is not applied along easy axis, the critical field is determined by exchange plus anisotropy. Why the critical field for B//c is larger in the twisted case (comparing Fig. 2b,d)? Does it mean the exchange is enhanced ?

Response

The critical field when the field is applied along not-the-easy-axis is given by (2 exchange field + anisotropy field). Please see the discussion in our Supplementary Notes 1 and 2, or Eqs. (S9) and (S10). Thus, the larger critical field for B//c simply stems from the c axis being the hardest anisotropy axis in the system.

Intuitively, it makes sense that we need the largest magnetic field to saturate the magnetization along the hardest axis.

Point by point reply.

Reviewer #1 (Remarks to the Author):

I thank the authors for addressing my comments.

I raise two main points and one minor comment (see below). Based on the authors' response, I believe our understanding of the underlying process is aligned. I hope these comments help clarify the message for a broader audience.

Overall, I am happy to recommend publication in Nature Communications once the authors address and clarify these points.

Reply: We thank the reviewer for this very positive assessment of the revised manuscript and the recommendation for publication.

1. As a first point, in their response the authors argue that: (1) their model, in the absence of strain due to experimental constraints, is expected to exhibit a spatially homogeneous magnetic state, switching, and hysteresis; and (2) their spatial mapping (which shows local modifications) highlights the importance of strain and lattice reconstruction due to twisting, and is therefore consistent with the reviewer's comment.

The inhomogeneities in the experimental samples are not a critical issue (in my view), since such variations in local strain are unavoidable when working with mechanically exfoliated and transferred 2D materials. Beyond moiré reconstruction, the formation of wrinkles and local strain variations during the transfer process is, to some extent, inevitable.

However, my main concern is that the authors develop a model (which is perfectly valid), but this model is not directly applicable to their experimental dataset, where local variations and additional effects such as strain play an important role. Despite this, the authors use the experimental data to extract parameters based on their model. Therefore, these extracted values should be interpreted with caution.

It would be useful for the authors to clearly acknowledge and discuss this point in the manuscript.

Reply: We thank the reviewer for this careful and constructive comment. We fully agree that spatial inhomogeneities such as local strain variations and reconstruction effects are present in mechanically exfoliated and transferred 2D heterostructures, and that these may influence the local magnetic response.

Our model captures the physics of the main experimental observations. Our model does not contain or aim at a fully microscopic description of the system and therefore does not explicitly incorporate local strain variations. For this reason, the parameters extracted from fitting the experimental data should be interpreted with appropriate caution, as they represent effective quantities within the framework of our model rather than direct microscopic values. **This is now made clear in the manuscript text.**

2. As a second point, the authors claim that the layers behave as a monodomain (page 5. "Our twisted bilayer sample seems to manifest sharp monodomain Stoner-Wohlfarth switching and does not offer any evidence of domains"). However, based on the data in Fig. 4, at a field of, for example, −0.1 T (red curve), position 1 is in a different state compared to positions 2 and 3. Thus, it is not clear how the switching can be described as monodomain for the whole twisted bilayer sample. At single locations the system may be described as such (in particular, for position 1 with a very sharp transition), but not for the whole twisted heterostructure (in position 2 and 3 there are two processes occurring: a sharp energy change and a more gradual one).

In addition, the field values used in Fig. S11 to support the monodomain claim are not well chosen. A minor loop would likely appear in Fig. S11 if a maximum field B_max of, for example, −0.15 T were applied, but not

for fields > 0 T. Regarding Fig. S10, I agree that the switching appears consistent with a monodomain behavior for that location.

Thus, the experimental data do not seem to support a monodomain scenario across the entire twisted bilayer. Therefore, I do not find the claim of monodomain switching across all positions to be fully supported. That said, I do not consider this a critical issue for the manuscript; softening or removing this claim would not affect the main conclusions. However, from data show in Fig 4, it is experimentally clear that different spin-switching processes are taken place considering the whole twisted-bilayer, incompatible with a monodomain switch across the whole heterostructure.

Reply: We thank the reviewer for this careful and insightful comment. We agree that the spatially varying switching behaviour observed in Fig. 4 indicates that the monodomain description does not apply across the entire twisted bilayer device, we find it remarkable that we can observe monodomain like switching at several sample positions. In the end, this is a matter of lengthscales which we have estimated in Supplementary Note 3, which was added during the last revision.

In the revised manuscript, we have therefore modified the wording to clarify that the sharp switching observed at position 1 is consistent with a **monodomain-like** Stoner–Wohlfarth–type response **at specific locations**. However, at other spatial positions (e.g., positions 2 and 3), the response shows additional gradual components, indicating that the switching behaviour is not uniform across the full heterostructure. We have adapted the text accordingly.

Minor comment: I note that references 19 and 36 (Boix-Constant et al.) are duplicates, as are references 32 and 47 (Liu et al.).

Thank you for your comment. We have modified the references in our modified manuscript.(implemented in the manuscript)

Reviewer #2 (Remarks to the Author):

I appreciate the authors' responses addressing my precious comments. However, the most critical point, which was raised by Reviewer#1, is that all data for the twisted crsbr were acquired from only a SINGLE device with 3degree twist. This raises severe concerns that the observed hysteresis highlighted in the main claim may just originate from extrinsic factors like strain or stacking faults, rather than moire-modulated exchange/anisotropy. Without angle dependent experiments and reproducible devices, their claim would remains unsubstantiated. Therefore, I cannot recommend its publication until such supportive experimental evidence is provided.

Answer:

Reply: We thank the reviewer for the response. We agree that establishing the intrinsic origin of the observed hysteresis is crucial.

In addition to the ~3° twisted monolayer–monolayer device presented in the main text, we have performed further measurements on a new ~2° twisted bilayer–bilayer device. This new device also exhibits clear magnetic hysteresis, absent in the pristine test sample. The observation of clear hysteretic behaviour across different twist angles and layer configurations supports the conclusion that the effect is intrinsic and arises from twist-induced modulation of the interlayer exchange.

We have now included these additional results on the new device in the revised manuscript (see Supplementary Note 8) which strengthen the reproducibility and generality of our findings.

03 June 2026

A point-by-point response to the reviewers' comments:

Reviewer #2 (Remarks to the Author):
“The authors have addressed all my concerns, and I'm happy to recommend the manuscipt for publication in Nat. Commun.”

We are happy the reviewers now recommend publication in Nature Communications.

For all the authors

Akashdeep Kamra and Bernhard Urbaszek
